# Disrupting the Hofmeister bias in salt liquid–liquid extraction with an arylethynyl bisurea anion receptor†

**DOI:** 10.1039/d3sc05922g

**Published:** 2024-03-07

**Authors:** Hazel A. Fargher, Lætitia H. Delmau, Vyacheslav S. Bryantsev, Michael M. Haley, Darren W. Johnson, Bruce A. Moyer

**Affiliations:** a Department of Chemistry and Biochemistry, Materials Science Institute, University of Oregon Eugene OR 97403-1253 USA haley@uoregon.edu dwj@uoregon.edu; b Radioisotope Science and Technology Division, Oak Ridge National Laboratory Oak Ridge TN 37831-6384 USA; c Chemical Sciences Division, Oak Ridge National Laboratory Oak Ridge TN 37831-6119 USA moyerba@ornl.gov

## Abstract

Host-mediated liquid–liquid extraction is a convenient method for the separation of inorganic salts. However, selective extraction of an anion, regardless of its hydrophilicity or lipophilicity as qualitatively described by its place in the Hofmeister series, remains challenging. Herein we report the complete disruption of the Hofmeister-based ordering of anions in host-mediated extraction by a rigidified tweezer-type receptor possessing remarkably strong anion-binding affinity under the conditions examined. Experiments introduce a convenient new method for determination of anion binding using phosphorus inductively coupled plasma mass spectrometry (ICP-MS) to measure extraction of tetra-*n*-butylphosphonium (TBP^+^) salts from water into nitrobenzene, specifically examining the disrupting effect of the added arylethynyl bisurea anion receptor. In the absence of the receptor, the salt partitioning follows the expected Hofmeister-type ordering favoring the larger, less hydrated anions; the analysis yields the value −24 kJ mol^−1^ for the standard Gibbs energy of partitioning of TBP^+^ cation from water into nitrobenzene at 25 °C. Selectivity is markedly changed by the addition of receptor to the nitrobenzene and is concentration dependent, giving rise to three selectivity regimes. We then used SXLSQI liquid–liquid equilibrium analysis software developed at Oak Ridge National Laboratory to fit host-mediated extraction equilibria for TBP^+^ salts of Cl^−^, Br^−^, I^−^, and NO_3_^−^ to the distribution data. While the reverse-Hofmeister 1 : 1 binding of the anions by the receptor effectively cancels the Hofmeister selectivity of the TBPX partitioning into nitrobenzene, formation of unexpected 2 : 1 receptor : anion complexes favoring Cl^−^ and Br^−^ dominates the selectivity at elevated receptor concentrations, producing the unusual order Br^−^ > Cl^−^ > NO_3_^−^ > I^−^ in anion distribution wherein a middle member of the series is selected and the most lipophilic anion is disfavored. Density functional theory calculations confirmed the likelihood of forming 2 : 1 complexes, where Cl^−^ and Br^−^ are encapsulated by two receptors adopting energetically competitive single or double helix structures. The calculations explain the rare non-Hofmeister preference for Br^−^. This example shows that anion receptors can be used to control the selectivity and efficiency of salt extraction regardless of the position of the anion in the Hofmeister series.

## Abstract 2

Host-mediated liquid–liquid extraction is a convenient method for the separation of inorganic salts.

## Graphical abstract

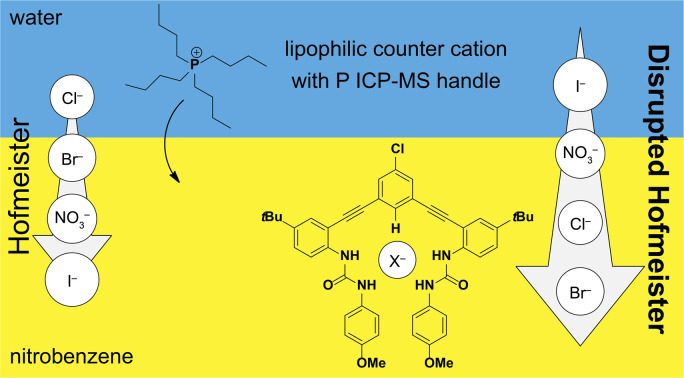

## Introduction

Separation of inorganic ions from complex solutions is a major challenge,^1^ with applications in mining,^2^ environmental remediation,^3,4^ resource recovery,^5^ nuclear-fuel recycle,^6,7^ and waste treatment,^8^ to name just a few. Advancing this field, host-mediated liquid–liquid extraction (LLE) has emerged as a powerful approach to impart enhanced selectivity and high binding affinities for desired guest ions.^9–12^ Research into host-mediated LLE has explored the influence of receptors on cation,^13^ anion,^14^ and ion-pair^15^ extraction. A significant body of this work has focused on the development of cation receptors, whereas complementary studies on anion receptors have lagged in comparison.^10^ In the absence of an anion receptor, the selectivity of partitioning from water into an organic phase is biased toward anions with increasing size or decreasing charge density, owing to the dominance of hydration energy *vs.* weaker solvation afforded by typical water-immiscible organic solvents.^16,17^ Thus, one obtains the hydration-based ordering originally noticed by Hofmeister,^18^ which persists widely in diverse chemical processes controlled by ion hydration.^19^ While Hofmeister-type selectivity is extremely useful, it has been particularly challenging to achieve desirable selectivity for small, highly hydrated anions in LLE. Even in the presence of anion receptors, one most often observes attenuated^20^ or perturbed^21,22^ Hofmeister ordering, as can be further influenced positively or negatively by ion-pairing. Given that most design strategies for anion receptors rely on hydrogen-bond donor groups, which in LLE must compete with the strong hydrogen-bonding received by the anion in the aqueous phase, extraordinary selectivity and affinity are needed. Accordingly, we recognize that overcoming the Hofmeister bias in LLE requires maximizing strong, complementary, and preorganized donor groups, in line with now-classical principles.^23,24^ Herein we present a case in which a rigidified tweezer-type receptor neatly fulfills these requirements, using novel methodology to show how LLE selectivity transitions from normal- to disrupted-Hofmeister behavior in the tug of war between the selectivity of anion partitioning and anion binding.

**Fig. 1:**
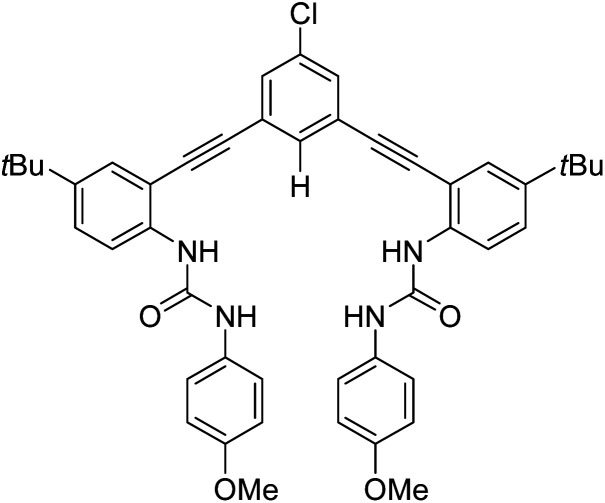
Anion receptor **R** used in this study.

We have introduced a family of arylethynyl bisurea receptors and studied their anion binding ability in homogenous organic solutions.^25–29^ The rigidity of the arylethynyl pivot is thought to impart greater anion binding affinity and selectivity in this tweezer-type receptor, which also features an active C–H donor group to complement its four N–H donors. We have also shown that the arylethynyl urea scaffold can be used in the design of receptors capable of selective extraction of hydrogen sulfate from sulfuric acid.^30^ Now we hypothesize that our receptors can be used to disrupt the Hofmeister type selectivity in LLE. Within the general framework of ways to deploy host-mediated LLE,^10^ this idea has previously been illustrated in so-called dual-host^31^ salt extraction (combination of cation and anion receptors) or synergized anion-exchange^21^ (combination of anion receptor with anion exchanger) systems. While it has been possible to use anion receptors alone to effect weak salt partitioning with inorganic cations in special cases,^21^ strong salt extraction is readily achievable with the use of sufficiently lipophilic cations such as long-chain quaternary ammoniums.^21,32^ To test our hypothesis, we use established arylethynyl bisurea anion receptor **R** (Fig. 1)^33,34^ with the lipophilic tetrabutylphosphonium (TBP^+^) cation to provide a novel handle for measuring salt extraction *via* phosphorus inductively coupled plasma mass spectrometry (ICP-MS).

## Methods

### Approach

Anion receptor **R** was chosen for this study due to its high anion-binding affinity in organic solvents, simple expected 1 : 1 host : guest binding stoichiometry, and negligible water solubility.^33,34^ The magnitude of the partition ratio of **R** between the organic and aqueous phases is estimated to be 1.1 × 10^10^ by the calculated log *P* function in ChemDraw,^35^ which is excessively sufficient to ensure that the exceedingly small fraction of **R** partitioned to the aqueous phase can be neglected. For this study, **R** was synthesized according to previously reported methods.^33^ TBP salts (TBPX, where X^−^ = Cl^−^, Br^−^, I^−^, and NO_3_^−^) were chosen for extraction experiments so that the change in aqueous P concentrations before and after extraction, measured by ICP-MS, could be used to measure salt partitioning. TBP salts were synthesized by reaction of aqueous TBPOH with the corresponding acid (HCl, HBr, HI, HNO_3_) and characterized by ^31^P NMR spectroscopy. Density functional theory (DFT) calculations were conducted to validate the possibility of forming stable 2 : 1 receptor : anion complexes with Cl^−^ and Br^−^. Further details on syntheses, materials, instrumentation, equipment, and DFT calculations are described in ESI.†

### Liquid–liquid extraction experiments

As detailed in ESI,† aqueous solutions containing variable concentrations of TBPX (0.02–0.03 mM, determined by ICP-MS) were equilibrated with equal volumes of purified nitrobenzene containing variable concentrations of **R** (2, 1.5, 1, 0.5, 0.1, 0.07, 0.05, and 0 mM). The maximum concentration of 2 mM was constrained by the solubility of **R** in nitrobenzene (<5 mM). After centrifugation, samples of the aqueous phases were removed and analyzed for P by ICP-MS before ([TBP]_0_) and after ([TBP]_aq_) equilibration. The distribution ratio *D*_P_, defined as [TBP]_org_/[TBP]_aq_, was then determined, where [TBP]_org_ is known by mass balance from the difference [TBP]_0_ − [TBP]_aq_.

## Results and discussion

### General equilibrium model

**Fig. 2:**
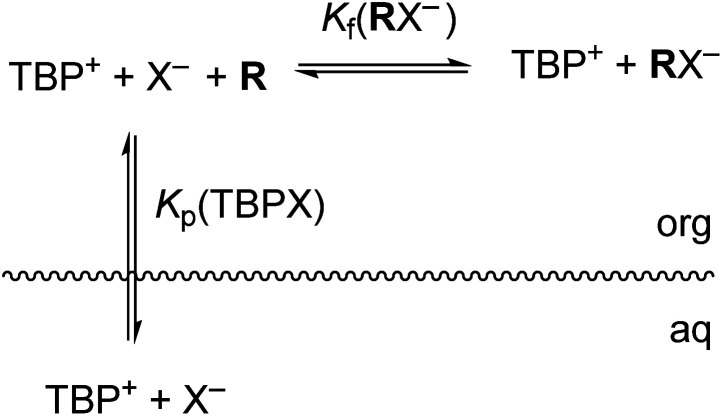
Equilibria present in host-mediated LLE of TBPX salts. Only a 1 : 1 **R**X^−^ complex is shown for simplicity, though other stoichiometries are possible.

The host-mediated LLE system investigated herein can be represented by a simple model given by the equilibria depicted in Fig. 2. Shown is salt partitioning of TBP^+^ cation and anion X^−^ from the aqueous to the organic phase, followed by complexation of the anion by **R**. Polar nitrobenzene provides for complete ion-pair dissociation in the organic phase at the low concentrations used, simplifying both analysis and understanding of binding. For simplicity of illustration consistent with our initial hypothesis, we include here only the anion host–guest complex with 1 : 1 binding stoichiometry with the understanding that general complex stoichiometries (**R**_*m*_X_*n*_^−^) can be accommodated (as in fact found necessary) in a straightforward, albeit more complicated, manner. The equations defining equilibrium constants are given in ESI.† The overall host-mediated extraction constant (*K*_ex±_) is the product of the salt partitioning constant (*K*_p_) and the formation constant (*K*_f_) of anion binding with host **R** (eqn (1)). *K*_ex±_ and *K*_p_ are determined directly from experiment, from which *K*_f_ is derived.

1 *K*_ex±_(TBP**R**X) = *K*_p_(TBPX) × *K*_f_(**R**X^−^)

### Salt partitioning equilibria

**Fig. 3:**
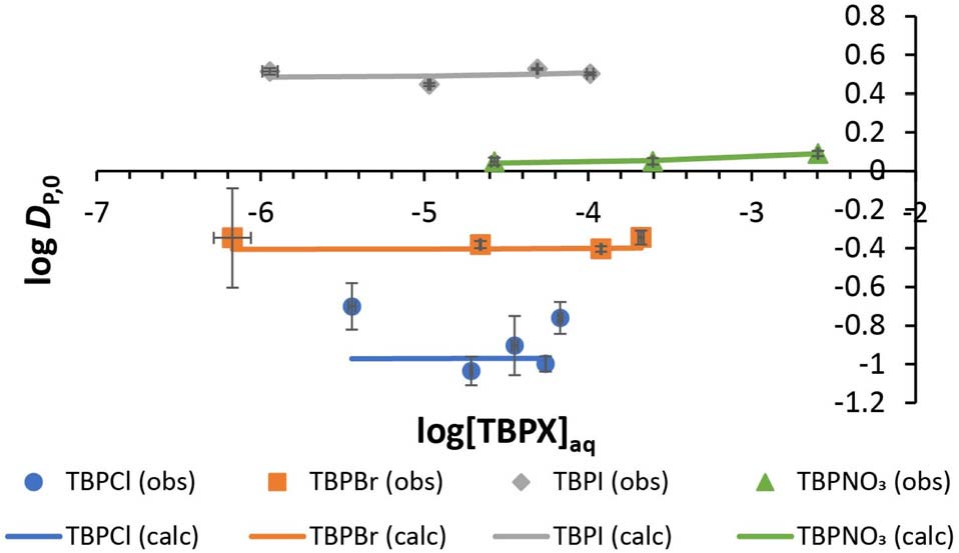
Experimental and calculated log *D*_P,0_*vs.* equilibrium log[TBPX]_aq_ for TBPCl, TBPBr, TBPI, and TBPNO_3_. Solid lines represent the calculated behavior using SXLSQI (*vide infra*).

Normal Hofmeister-type selectivity was observed in LLE of TBPX salts by nitrobenzene without added **R**. As shown in Fig. 3, the obtained four plots of salt distribution ratios expressed as log *D*_P,0_*vs.* aqueous salt concentration at equilibrium ([TBPX]_aq_) follow the order I^−^ > NO_3_^−^ > Br^−^ > Cl^−^. Corresponding data for liquid–liquid extractions of 0.0014–12.62 mM TBPX salts are given in Tables S1–S4.† A flat dependence is observed as expected (eqn (S7)†), where a slight activity-coefficient-induced curvature at the higher TBPX concentrations is captured by equilibrium analysis (solid lines in Fig. 3) using the program SXLSQI^36,37^ (Solvent eXtraction Least SQuares—Ion; see ESI†). The analysis yields corresponding equilibrium constants (log *K*_p_) as given in Table 1. Given the known values of the standard Gibbs energies of anion partitioning, 
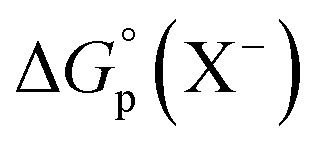
,^38^ the previously unknown value of 
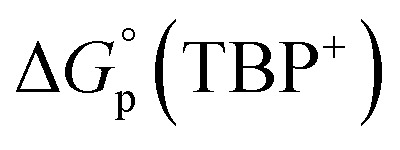
 was found to be −24 ± 1 kJ mol^−1^, revealing the push–pull dynamics of favorable cation partitioning overcoming unfavorable anion partitioning. Within experimental error, the observed value of 
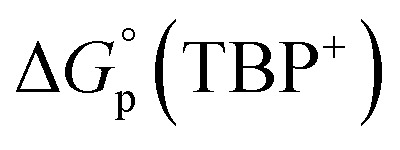
 turns out to be the same as that for the more commonly used tetra-*n*-butyl ammonium cation.^39^

**Table 1:** Experimental values of log *K*_p_ for TBPCl, TBPBr, TBPI, and TBPNO_3_ partitioning from water into nitrobenzene at 25 °C calculated by SXLSQI and determination of the standard Gibbs energy of TBP^+^ partitioning, 
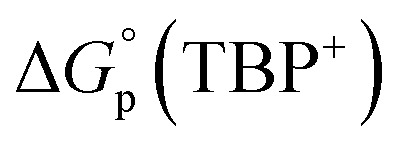
a

Salt	Average log *K*_p_	Equilibrium	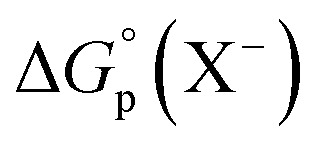 ^40^ (kJ mol^−1^)	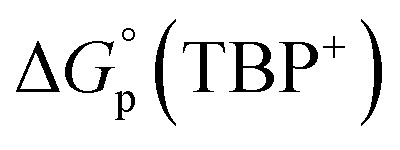 (kJ mol^−1^)
TBPCl	−1.94 ± 0.09	TBP^+^_(aq)_ + Cl^−^_(aq)_ ⇌ TBP^+^_(org)_ + Cl^−^_(org)_	35	−24 ± 1
TBPBr	−0.81 ± 0.02	TBP^+^_(aq)_ + Br^−^_(aq)_ ⇌ TBP^+^_(org)_ + Br^−^_(org)_	29	−24 ± 1
TBPI	0.97 ± 0.03	TBP^+^_(aq)_ + I^−^_(aq)_ ⇌ TBP^+^_(org)_ + I^−^_(org)_	18	−24 ± 1
TBPNO_3_	0.070 ± 0.007	TBP^+^_(aq)_ + NO_3_^−^_(aq)_ ⇌ TBP^+^_(org)_ + NO_3_^−^_(org)_	24	−24 ± 1
			Average =	−24 ± 1

a Single-ion standard Gibbs energies of partitioning shown here use the TATB (tetraphenylarsonium tetraphenylborate) extrathermodynamic assumption.^40^ Values of log *K*_p_ were converted to corresponding Gibbs energies according to 
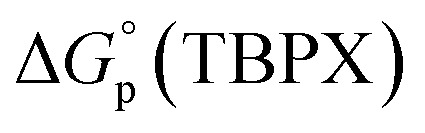
 = −2.303*RT* log *K*_p_. See ESI for full discussion.

### Host-mediated extraction disrupts Hofmeister selectivity

#### General behaviour

**Fig. 4:**
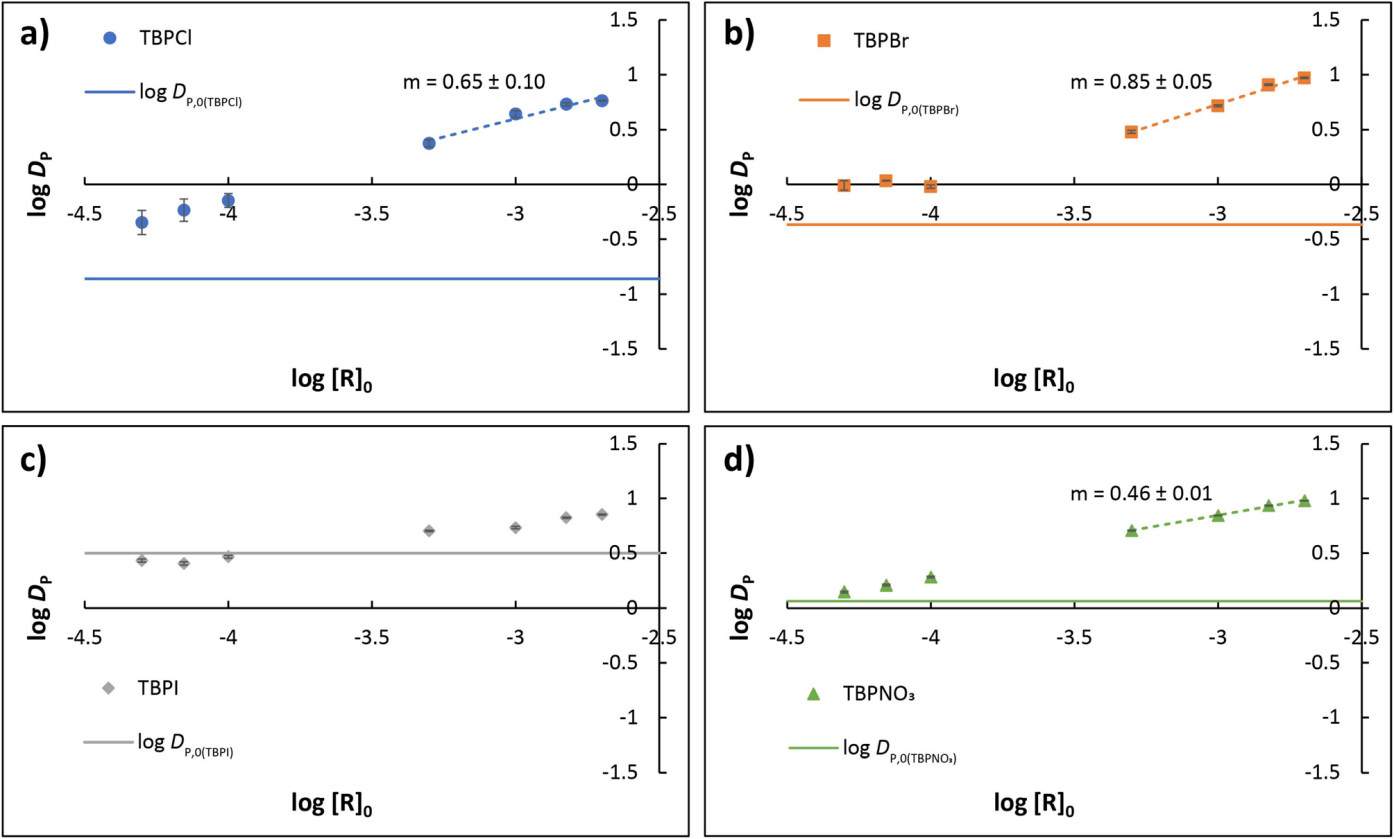
Experimentally determined log *D*_P_ plotted as a function of log[**R**]_0_ for (a) TBPCl, (b) TBPBr, (c) TBPI, and (d) TBPNO_3_, where [**R**]_0_ is the intitial concentration of **R**. Solid lines demark *D*_P,0_ for each salt. Linear dashed segments denote an approximate slope analysis, which suggests formation of a 1 : 1 anion complex **R**X^−^ for nitrate with mixed 1 : 1 and 2 : 1 species **R**_*m*_X^−^ for chloride and bromide according to predicted slope 0.5*m*. Enhancement for TBPI is insufficient for slope analysis. (See ESI†).

Addition of host **R** markedly enhances the distribution of anions to the organic phase. Liquid–liquid extractions of a fixed initial concentration of TBPX salts in the range 0.02–0.03 mM were performed with nitrobenzene at 25 °C containing various concentrations of host **R** (2, 1.5, 1, 0.5, 0.1, 0.07, and 0.05 mM) (Tables S5–S8†). Experimental values of log *D*_P_ are plotted against log[**R**]_0_ for Cl^−^, Br^−^, I^−^, and NO_3_^−^ in Fig. 4a–d, respectively; see ESI† for additional plots (Fig. S11†). With decreasing values of the host concentration, log *D*_P_ asymptotically approaches the baseline values of log *D*_P,0_ shown for each salt in Fig. 3 (demarked by solid lines in Fig. 4a and b). With increasing host concentration, however, extraction for all anions becomes more favorable, and at the highest initial **R** concentration (2 mM), an obviously disrupted Hofmeister bias emerges in terms of overall extraction strength (*D*_P_): NO_3_^−^ (9.54 ± 0.06) ∼ Br^−^ (9.4 ± 0.1) > I^−^ (7.11 ± 0.08) > Cl^−^ (5.80 ± 0.06).

#### Analysis of selectivity in host-mediated extraction of TBPX

Rigorous equilibrium analysis of the host-mediated extraction data implies formation of both 1 : 1 and 2 : 1 **R**_*m*_X^−^ complexes. While the simple model shown in Fig. 2 hypothesized a 1 : 1 host : anion binding stoichiometry, the dashed-line behavior shown in Fig. 4 suggests that 2 : 1 binding is also important for chloride and bromide, with special implications for selectivity (see ESI†). More exact modeling of the extraction data using the SXLSQI liquid–liquid equilibrium analysis software^36,37^ corroborated the qualitative insight from slope analysis and yielded extraction constants for all four anions, including iodide. Equilibria for the host-mediated models considered most valid for each anion are shown together with corresponding calculated log *K*_ex±_ in Table 2. The complete set of data (Tables S1–S8†) was treated, estimating activity coefficients for species in both phases in the calculation using parameters given in Tables S11–S14.† Plots comparing observed and calculated points are shown in Fig. S12–S15.†

**Table 2:** Determined extraction constants (log *K*_ex±_) using SXLSQI for host-mediated extraction of TBPCl, TBPBr, TBPI, and TBPNO_3_ from water into nitrobenzene at 25 °C^a^

TBPX	Equilibrium	Log *K*_ex±_
TBPCl	TBP^+^_(aq)_ + Cl^−^_(aq)_ + **R**_(org)_ ⇌ TBP^+^_(org)_ + **R**Cl^−^_(org)_	3.9 ± 0.2
	TBP^+^_(aq)_ + Cl^−^_(aq)_ + 2**R**_(org)_ ⇌ TBP^+^_(org)_ + **R**_2_Cl^−^_(org)_	6.7 ± 0.2
TBPBr	TBP^+^_(aq)_ + Br^−^_(aq)_ + **R**_(org)_ ⇌ TBP^+^_(org)_ + **R**Br^−^_(org)_	4.17 ± 0.06
	TBP^+^_(aq)_ + Br^−^_(aq)_ + 2**R**_(org)_ ⇌ TBP^+^_(org)_ + **R**_2_Br^−^_(org)_	7.20 ± 0.07
TBPI	TBP^+^_(aq)_ + I^−^_(aq)_ + **R**_(org)_ ⇌ TBP^+^_(org)_ + **R**I^−^_(org)_	4.34 ± 0.06
TBPNO_3_	TBP^+^_(aq)_ + NO_3_^−^_(aq)_ + **R**_(org)_ ⇌ TBP^+^_(org)_ + **R**NO_3_^−^_(org)_	4.65 ± 0.03

a Observed values of *D*_P_ were taken from Tables S5–S8, yielding the corresponding output values of refined log *K*_ex±_. In the fitting of the extraction data for each anion, the corresponding log *K*_p_ values were fixed at the values shown in Table 1 to account for the background extraction of TBPX by nitrobenzene alone.

**Fig. 5:**
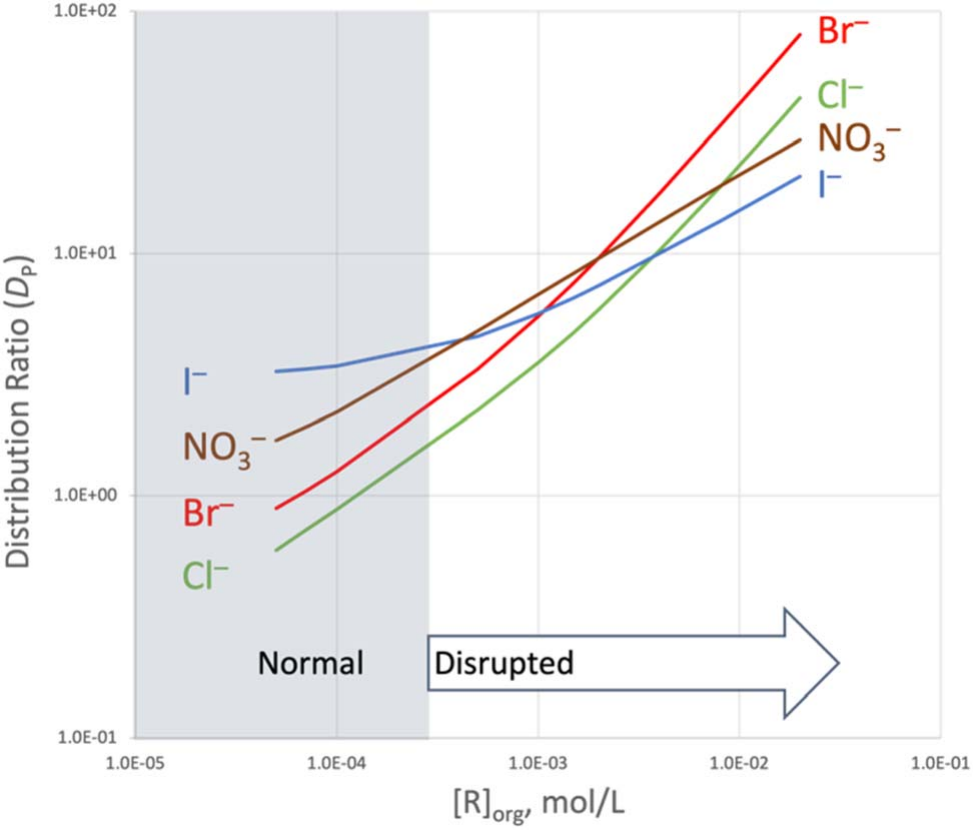
Comparison of calculated values of *D*_P_ for extraction of the four TBP salts as a function of [**R**]. The plotted domain 2 mM < [**R**] ≤ 20 mM represents an extrapolation. Solid lines were calculated using SXLSQI based on the model values of log *K*_p_ (Table 1) and log *K*_ex±_ (Table 2).

Using the equilibrium constants given in Tables 1 and 2 to calculate smoothed distribution profiles illustrates the progression from normal to disrupted Hofmeister ordering (Fig. 5). Dominant partitioning of TBPX yields normal Hofmeister behavior at low concentration of **R**, giving way to disrupted Hofmeister ordering controlled by host-mediated extraction. At mid concentrations of **R**, the distribution ratios tend to converge with five crossover points. As the steepness of the chloride and bromide curves increases owing to the onset of 2 : 1 **R**_2_X^−^ complex formation, the distribution ratios diverge, giving rise to the order Br^−^ > Cl^−^ > NO_3_^−^ > I^−^ at the highest plotted concentrations. Selection of a middle member of the Hofmeister series is quite unusual in LLE systems as is rejection of the largest, most lipophilic anion in the series.

#### Understanding factors underlying selectivity in host-mediated extraction of TBPX

Comparing extraction constants for all salts reveals the roles of 1 : 1 and 2 : 1 binding in disrupting Hofmeister selectivity. From the log *K*_ex±_ values shown in Table 2 for formation of 1 : 1 complexes, one may see that the Hofmeister ordering is largely canceled by the anion host. The rendered order NO_3_^−^ > I^−^ > Br^−^ > Cl^−^ of log *K*_ex_ for 1 : 1 complexes is weak, with a spread of only 0.7 log units *vs.* a spread of 2.0 log units for salt partitioning (Table 1). The propensity of chloride and bromide to form 2 : 1 complexes proves decisive at higher receptor concentrations, where the corresponding 2 : 1 log *K*_ex±_ values are three log units higher than the corresponding 1 : 1 log *K*_ex±_ values. Extrapolating the model to 20 mM receptor (Fig. 5) accentuates the effect of the 2 : 1 binding on *D*_P_.

Binding constants provide further insight. More familiar to coordination chemists, anion binding constants log *K*_f_ for each salt with **R** are obtained here by subtracting log *K*_p_ from log *K*_ex±_. We have generally found that binding constants determined by rigorous analysis of LLE data match those obtained by other methods (for example, see ref. 31). These results are shown in Table 3. It may be seen that the 1 : 1 binding constants follow the reversed-Hofmeister order of extraction Cl^−^ > Br^−^ > NO_3_^−^ > I^−^. Flexible anion receptors often follow this trend, where the hydrogen bond strength increases with decreasing anion size or increasing charge density. Although **R** binds Cl^−^ an order of magnitude more favorably than Br^−^, the binding of a second **R** with the **R**X^−^ complex is statistically at least as large for Br^−^ as it is for Cl^−^.

**Table 3:** Derived formation constants (log *K*_f_) for anion binding by **R** in water-saturated nitrobenzene as inferred from host-mediated extraction of TBPCl, TBPBr, TBPI, and TBPNO_3_ from water into nitrobenzene at 25 °C^a^

TBPX	Equilibrium	Log *K*_f_
TBPCl	**R**_(org)_ + Cl^−^_(org)_ ⇌ **R**Cl^−^_(org)_	5.8 ± 0.2
	2**R**_(org)_ + Cl^−^_(org)_ ⇌ **R**_2_Cl^−^_(org)_	8.6 ± 0.1
	**R**_(org)_ + **R**Cl^−^_(org)_ ⇌ **R**_2_Cl^−^_(org)_	2.8 ± 0.2
TBPBr	**R**_(org)_ + Br^−^_(org)_ ⇌ **R**Br^−^_(org)_	4.98 ± 0.05
	2**R**_(org)_ + Br^−^_(org)_ ⇌ **R**_2_Br^−^_(org)_	8.02 ± 0.07
	**R**_(org)_ + **R**Br^−^_(org)_ ⇌ **R**_2_Br^−^_(org)_	3.03 ± 0.09
TBPI	**R**_(org)_ + I^−^_(org)_ ⇌ **R**I^−^_(org)_	3.78 ± 0.05
TBPNO_3_	**R**_(org)_ + NO_3_^−^_(org)_ ⇌ **R**NO_3_^−^_(org)_	4.58 ± 0.03

a Values of log *K*_f_ were obtained by subtracting values log *K*_p_ from log *K*_ex±_ for each salt taken from Tables 1 and 2, respectively.

Structural evidence provides clues regarding the observed 1 : 1 and 2 : 1 anion binding stoichiometries. Previous DFT calculations for 1 : 1 complexes reveal that the O-atoms of nitrate are accommodated in an approximately planar array of the available N–H and C–H donors of the arylethynyl host structure.^33^ In accord with criteria described for nitrate binding by urea hydrogen-bond donors,^40^ the two urea groups of the host bind along edges of the nitrate anion. The saturation of the nitrate coordination sites by a single **R** molecule logically explains the tendency of the stoichiometry to remain 1 : 1. By contrast, an X-ray structure shows a non-planar configuration of the urea donor groups for 1 : 1 binding of the smaller chloride anion, one urea twisted below and one above the plane of the central benzene ring in an opposing manner. Chloride can structurally accommodate four urea donor groups,^40^ lending a structural basis for 2 : 1 binding of **R** to chloride or bromide. Possibly iodide would exhibit 2 : 1 binding in this way, but solubility limitations prevented our testing sufficiently high **R** concentrations. We believe that the lack of ion pairing in nitrobenzene adds another factor favoring 2 : 1 binding. Anion binding by **R** and its various modifications was studied previously using chloroform as the solvent,^33^ where the much lower dielectric constant (*ε* = 4.81)^46^ promotes ion pairing. Displacement of the associated cation in the C^+^[**R**X^−^] complex ion pair by a second molecule of **R** represents an electrostatic energy penalty, weakening its binding. Experimentally, three-fold lower concentrations of **R** were used in the reported NMR titrations, and high residuals were seen in the early points of the data fits,^33^ suggesting some formation of 2 : 1 species had still occurred.

**Fig. 6:**
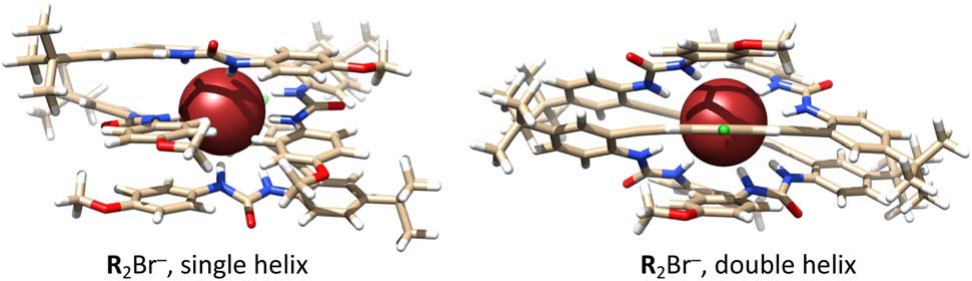
Optimized molecular structures of the complexes with Br^−^ forming the single helix (left) and double helix (right) of **R** using DFT and implicit solvent model for nitrobenzene.

DFT calculations supported the formation of 2 : 1 complexes with Cl^−^ and Br^−^ implied by the equilibrium analysis and additionally shed light on the unusual selectivity observed for Br^−^. The structural modification of 1 : 1 complexes to allow for the inclusion of two ligands in either a ‘sandwich’ or intertwined configuration has yielded two unique assemblies exhibiting competitive energetics. As shown in Fig. 6, two receptors are symmetrically arranged around each anion, resulting in the formation of either a single or a double helicate structure. Thermodynamic calculations (Table S16†) for **R**_(org)_ + **R**X^−^_(org)_ ⇌ **R**_2_X^−^_(org)_ (X = Cl^−^ and Br^−^) confirmed the stability of the 2 : 1 complexes, both in the gas phase and with the inclusion of an implicit solvent (nitrobenzene). We find that the formation of the 2 : 1 complexes with Br^−^ is slightly more favorable than with Cl^−^, which is uncommon, as the more charge-diffuse Br^−^ ion is expected to form weaker bonds with hydrogen bond donors. Examination of hydrogen bond distances, as detailed in Table S17,† shows that both Cl^−^ and Br^−^ strongly interact with the four urea groups in the two ligands. Yet, the extent to which the hydrogen bonds are elongated compared to the those in the 1 : 1 complexes is slightly larger for Cl^−^ compared to Br^−^. This result aligns with the geometric patterns of binding, as the larger Br^−^ ion offers greater spatial accessibility for binding in the tight assembly of two receptors.

Finally, we note the remarkably high binding constants determined for all anions with **R**, which we ascribe to structural rigidity as well as solvent effects. Although a reorganization penalty typically weakens host–guest complexation in flexible hosts, the conjugated system of our receptor imparts significant rigidity compared with many tweezer-type hosts, likely reducing the effect of this penalty. In addition, we have previously suggested that shape-persistent hosts may experience a greater solvation penalty and a greater ground-state destabilization in more polar solvents, compared with flexible hosts.^41^ The properties of the nitrobenzene diluent used here also favor stronger binding. In Table 4 we compare log *K*_f_ for 1 : 1 binding in nitrobenzene (dielectric constant *ε* = 34.82)^42^ to binding constants of the same host in H_2_O_sat._ CHCl_3_ (*ε* ≈ 4.9)^43^ and 10% DMSO-*d*_6_/CD_3_CN (*ε* ≈ 42).^43^ Although solvent-system polarity is often a predictor of binding strength,^42,44^**R** binds all anions much more favorably in nitrobenzene compared to the two other solvent systems, irrespective of the solvent system dielectric strength. The hydrogen bond accepting and donating ability of 10% DMSO-*d*_6_/CD_3_CN provides for solvation of the host and the anion guest, competing with anion binding.^45^ One of the strongest electron-pair donors among organic solvents,^44^ DMSO in particular would be expected to engage the hydrogen bond donor groups of **R**, in accord with the lowest binding constants observed in Table 4. As mentioned above, ion pairing in the case of water-saturated CHCl_3_ by the TBA^+^ counter cation impedes binding.

**Table 4:** Previously reported log *K*_f_ for 1 : 1 binding of the halides and nitrate with **R** in two different solvent systems compared with nitrobenzene at 25 °C

X^−^	H_2_O_sat._ CHCl_3_ (ref. 33)	10% DMSO-*d*_6_/CD_3_CN^34^	Nitrobenzene^b^
Cl^−^	3.90 ± 3.11	3.36 ± 2.26	5.8 ± 0.2
Br^−^	3.43 ± 2.36	2.1 ± 0.8	4.98 ± 0.05
I^−^	2.5 ± 1.0	a	3.78 ± 0.05
NO_3_^−^	3.74 ± 2.81	a	4.58 ± 0.03

a Value not measured in study.

b This work. See Table 2.

## Conclusions

This work confirms our hypothesis that a previously published arylethynyl bisurea receptor **R**^33,34^ with enhanced rigidity would disrupt the Hofmeister-type selectivity in LLE of anions. This host enhances the extraction of four anions as TBP^+^ salts into nitrobenzene at 25 °C. Normal Hofmeister-type extraction selectivity (I^−^ > NO_3_^−^ > Br^−^ > Cl^−^) progresses to an unusual disrupted Hofmeister selectivity (Br^−^ > Cl^−^ > NO_3_^−^ > I^−^) favoring a middle member of the series and rejecting the most lipophilic anion (Fig. 5). The distribution ratios for the TBPX salts were used to calculate equilibrium constants for extraction and binding using the SXLSQI equilibrium modeling. Based on the equilibrium constants, Table 5 summarizes the underlying influence of binding on LLE selectivity of the anions, categorizing the selectivity shift as the concentration of receptor increases. Partitioning follows the normal Hofmeister order. Binding at low host concentrations follows a reversed Hofmeister order for 1 : 1 stoichiometry, which largely cancels the normal Hofmeister order obtained at zero host concentration; however, the disrupted Hofmeister selectivity at the elevated host concentrations turns on unexpected 2 : 1 binding. DFT calculations corroborate the assembly of 2 : 1 complexes with Cl^−^ and Br^−^, indicating the possibility of forming single and double helical structures with **R** and explaining the selectivity for Br^−^. We consider the DFT support for the formation of 2 : 1 complexes *via* helicate assembly to be a breakthrough in the understanding of our rigidified tweezer molecules. To date, evidence for formation of 2 : 1 species has been scant, and no structural insight has been hitherto revealed, providing ample fodder for future investigation by, for example, adding electron-withdrawing groups or added structural constraints to the molecular structure to modulate selectivity.

**Table 5:** Selectivity of partitioning, binding, and host-mediated extraction of TBPX salts from water into nitrobenzene at 25 °C

Regime	Dominant equilibrium	Selectivity
Partitioning (no host)^a^	TBP^+^_(aq)_ + X^−^_(aq)_ ⇌ TBP^+^_(org)_ + X^−^_(org)_	I^−^ > NO_3_^−^ > Br^−^ > Cl^−^ (normal)
1 : 1 binding^b^	**R**_(org)_ + X^−^_(org)_ ⇌ **R**X^−^_(org)_	Cl^−^ > Br^−^ > NO_3_^−^ > I^−^ (reverse)
2 : 1 binding^b^	2**R**_(org)_ + X^−^_(org)_ ⇌ **R**_2_X^−^_(org)_	Cl^−^ > Br^−^ ≫ NO_3_^−^, I^−^ (disrupted)
Host-mediated extraction^c^	TBP^+^_(aq)_ + X^−^_(aq)_ + **R**_(org)_ ⇌ TBP^+^_(org)_ + **R**X^−^_(org)_	NO_3_^−^ > I^−^ > Br^−^ > Cl^−^ (weakly disrupted)
Host-mediated extraction^c^	TBP^+^_(aq)_ + X^−^_(aq)_ + 2**R**_(org)_ ⇌ TBP^+^_(org)_ + **R**_2_X^−^_(org)_	Br^−^ > Cl^−^ ≫ NO_3_^−^, I^−^ (disrupted)

a See Table 1 for log *K*_p_ values.

b See Table 3 for log *K*_f_ values.

c See Table 2 for log *K*_ex±_ values and Fig. 5 for comparison of distribution ratios *D*_P_ as a function of [**R**].

A useful by-product of this investigation is the introduction of experimental methodology for the convenient study of anion extraction and binding. The method employs ICP-MS instrumentation routinely available to many experimenters to follow the distribution of the TBP^+^ cation. The value −24 kJ mol^−1^ has been determined for its single-ion partitioning from water into nitrobenzene at 25 °C.

In general, development of selective anion receptors could be hugely beneficial in LLE applications, from improving extraction efficiencies,^17^ to targeting anions of interest,^11,12^ and even influencing cation selectivity.^47^ Except where Hofmeister selectivity is desired, advances will hinge on understanding and controlling the selectivity of anion binding. As we and others still appreciate, disrupted Hofmeister ordering in LLE is not often observed and exceedingly difficult to achieve,^20–22,31,48–51^ challenged especially as applications demand inexpensive materials. Despite voluminous progress on anion receptors and recognition,^52–56^ the field remains in a dynamic and exciting state of development.

## Data availability

Experimental methods, materials, raw data, data treatment, computational methods, and computational results are provided in ESI.†

## Author contributions

The manuscript was written through contributions of all authors. All authors have given approval to the final version of the manuscript.

## Conflicts of interest

There are no conflicts to declare.

## Supplementary Material

SC-015-D3SC05922G-s001
