# Supplementary material for: Disrupting the Hofmeister bias in salt liquid–liquid extraction with an arylethynyl bisurea anion receptor
Source: Chem Sci. 2024 Mar 7;15(14):5311–8. doi: 10.1039/d3sc05922g (PMC10988605; doi:10.1039/d3sc05922g)
Supplement: SC-015-D3SC05922G-s001 [file SC-015-D3SC05922G-s001.pdf]

Electronic Supplementary Material (ESI) for Chemical Science.
This journal is © The Royal Society of Chemistry 2024

Supplementary Information

Disrupting the Hofmeister Bias in Salt Liquid-Liquid Extraction with an Arylethynyl Bisurea Anion Receptor

Hazel A. Fargher,[a] Lætitia H. Delmau,[b] Vyacheslav S. Bryantsev,[c] Michael M. Haley,[a*] Darren W. Johnson, [a*] Bruce A. Moyer[c*]

[a] Department of Chemistry and Biochemistry, Materials Science Institute, and Knight Campus for Accelerating Scientific Impact, University of Oregon, Eugene, OR, USA

[b] Radioisotope Sciences and Technology Division, Oak Ridge National Laboratory, Oak Ridge, TN, USA

[c] Chemical Sciences Division, Oak Ridge National Laboratory, Oak Ridge, TN, USA

Email: haley@uoregon.edu, dwj@uoregon.edu, moyerba@ornl.gov

MATERIALS

General Methods

All reagents were purchased from commercial sources and used as received, unless otherwise noted. Nitrobenzene was purified according to previous methods.[1] 18 MΩ deionized water was obtained from a Milli-Q IQ 7000. All glassware was cleaned in an overnight acid bath and rinsed with 18 MΩ deionized water. Non-analytical glassware was also cleaned by overnight base bath. Receptor **R** was synthesized according to previous methods.[2] NMR spectra were acquired at room temperature on a Bruker Avance-III-HD 500 MHz (^{1}H 500 MHz, ^{31}P 202.46 MHz) spectrometer with a Prodigy multinuclear broadband BBO CryoProbe. ^{1}H chemical shifts (δ) are reported in ppm relative to CH_3CN (^{1}H: 1.94 ppm). ^{31}P chemical shifts are referenced to 85% H_3PO_4 (δ = 0 ppm) as an external standard.

General TBPX Synthesis

Commercial TBPOH was purified by serial recrystallization in anhydrous THF layered under anhydrous Et_2O, under an inert atmosphere using an Innovative Atmospheres N_2-filled glove box. Precipitate was filtered and dissolved in Milli-Q water to make a 40 mM solution. For each salt, 1.1 equivalents of the corresponding aqueous acid (conc. HCl, conc. HBr, conc. HI, conc. HNO_3; ACS reagent grade) were added to neutralize a 5 mL solution of 5 mM TBPOH in Milli-Q

water. The aqueous solution was extracted x3 with 5 mL DCM and the organic phase was evaporated to dryness. Yields were not obtained. ^{31}P NMR was used to check purity of phosphorous-containing species.

NMR Spectra of Synthesized TBPX

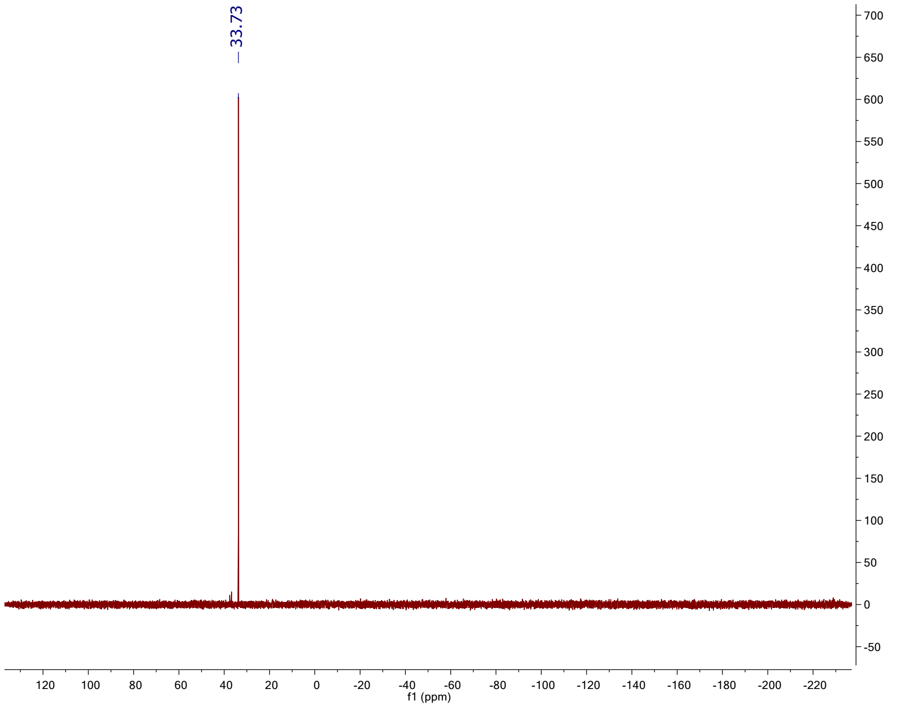

Figure S1. Spectra of TBPOH. ^{31}P NMR (202.46 MHz, CD_3CN) δ 33.73.

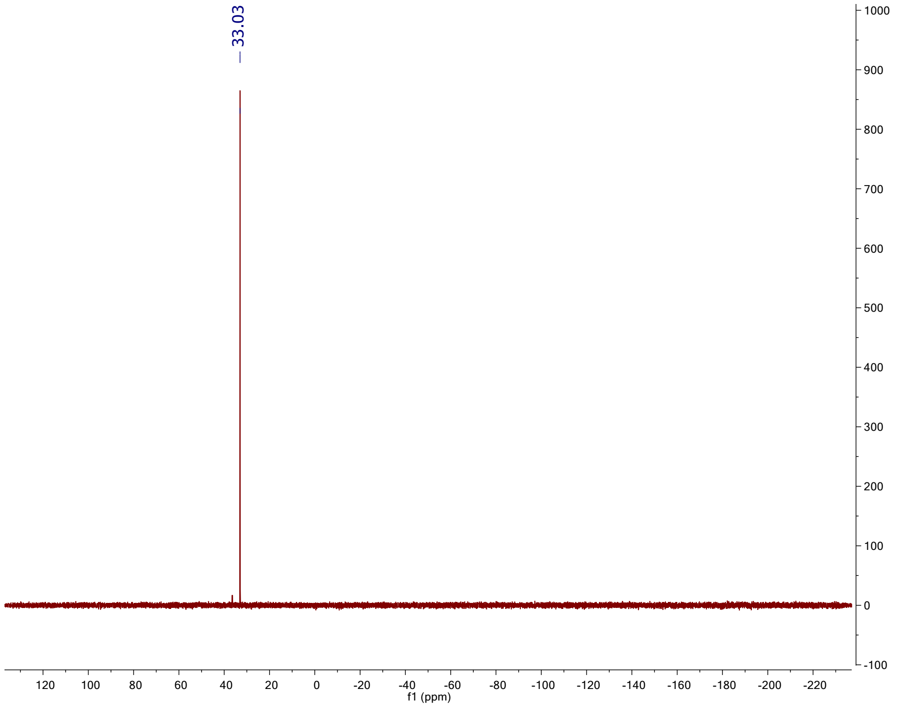

Figure S2. Spectra of TBPCl. ^{31}P NMR (202.46 MHz, CD_3CN) δ 33.03.

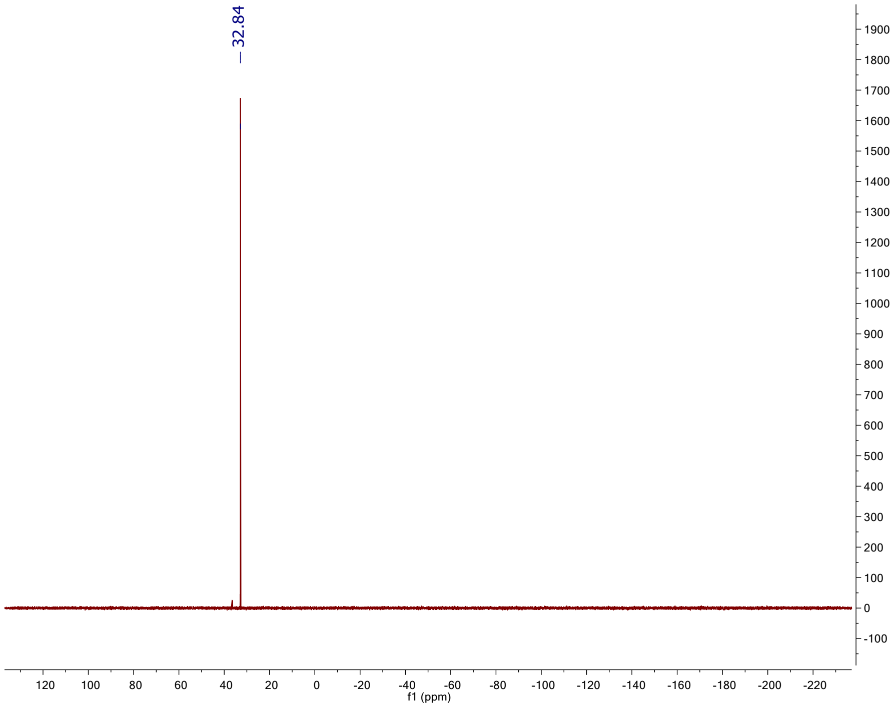

Figure S3. Spectra of TBPBr. ^{31}P NMR (202.46 MHz, CD_3Cl) δ 32.84.

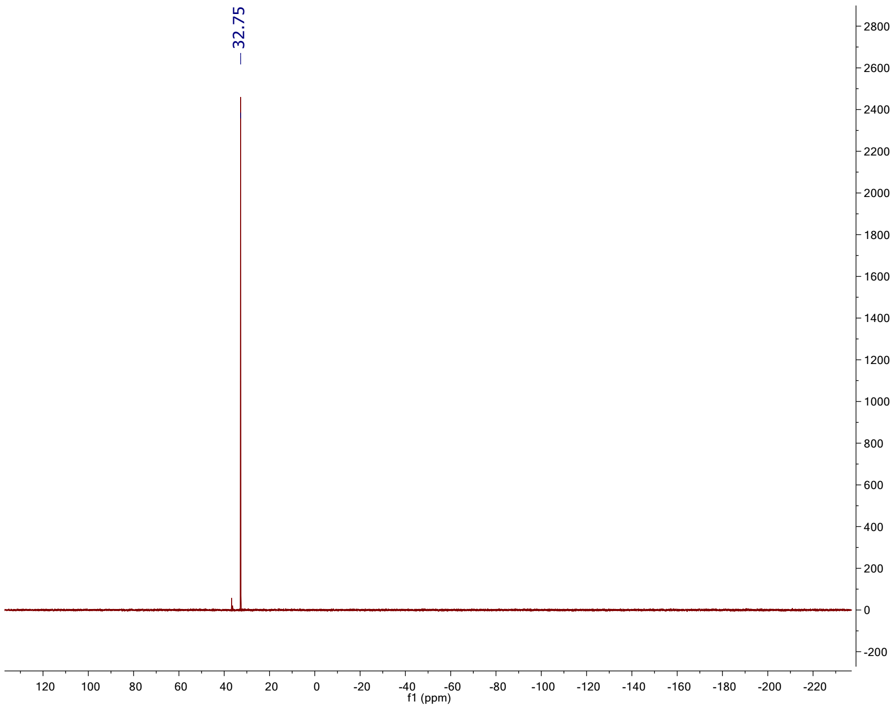

Figure S4. Spectra of TBPI. ^{31}P NMR (202.46 MHz, CD_3Cl) δ 32.75.

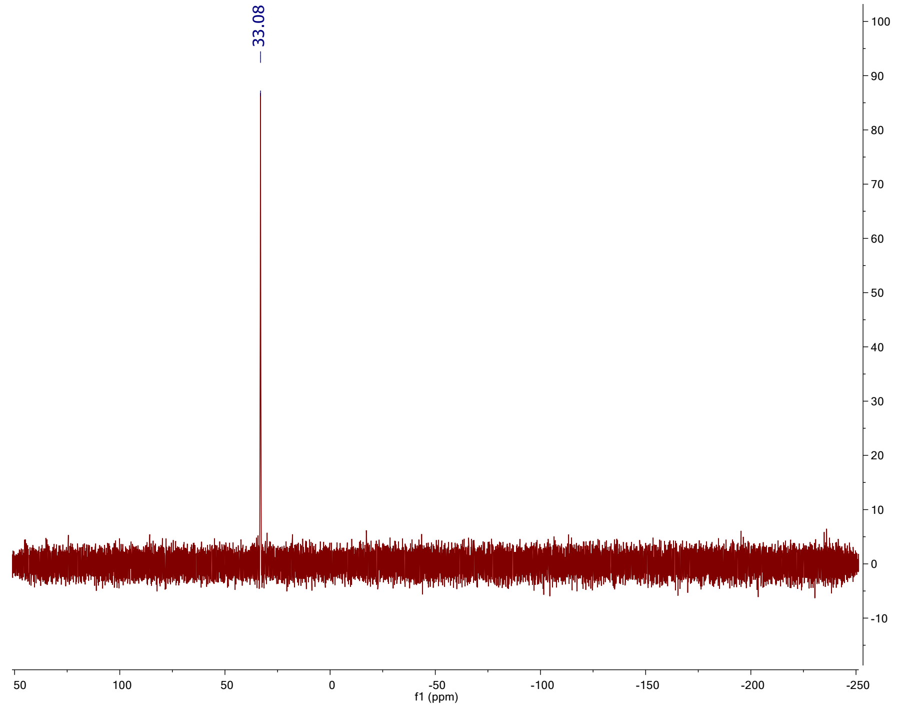

Figure S5. Spectra of $TBPNO_3$. ^{31}P NMR (202.46 MHz, CD_3Cl) δ 33.08.

EXTRACTION AND ANALYTICAL METHODS

Liquid-liquid Contacting Method

Aqueous solutions contained variable concentrations of TBPX (0.02–0.03 mM, determined by ICP-MS) in 18 MΩ deionized water. Organic solutions of purified nitrobenzene containing variable concentrations of **R** (2, 1.5, 1, 0.5, 0.1, 0.07, 0.05, and 0 mM) were contacted with equal volumes (0.3 mL each) of the aqueous solutions by repeated inversion at 8 RPM on a tube rotator for 2 h. After rotation, samples were centrifuged at 3000 RCF for 20 min at 25 °C in a temperature-controlled centrifuge. After centrifugation, 0.2 mL of the aqueous phase was removed and prepared for analysis by ICP-MS (see below). All extractions were performed in duplicate or triplicate and precision determined therefrom as the standard deviation.

Salt Extraction Quantification

TBPX salt extraction can be measured following either the cation distribution or the anion distribution. Since the organic cation is common to all experiments, here salt extraction is most conveniently quantified as the distribution ratio (D_P) of TBP^+ cations between the organic and aqueous phase at equilibrium (Equation S1). Phosphorus concentration in aqueous solution can be determined by ICP-MS with limits of detection in the ppt range and background equivalent concentrations in the ppb range (see below). However, the P concentration in the organic phase could not be directly determined through ICP-MS. Therefore, based on mass balance at unit phase-volume ratio, $[TBPX]_{org}$ was calculated by subtracting $[TBPX]_{aq}$ after extraction from the initial salt concentration before extraction ($[TBPX]_0$) (Equation S1). $[TBPX]_0$ was determined by

subjecting the original aqueous solution to the same experimental and sample preparation conditions as the contacting experiments, without the presence of an organic phase. Each $[TBPX]_0$ and the aqueous TBPX concentration after contact, $[TBPX]_{aq}$, is an average of values determined from duplicate or triplicate experiments. Standard error shown for $[TBPX]_0$ and $[TBPX]_{aq}$ is the standard deviation of the replicate trials. The distribution ratio of TBP^+ cations in the absence of host **R** is indicated by $D_{P,0}$. Using Equation S1 limits the obtainable values of D_P to not less than approximately 0.1 because the difference the difference $[TBP]_0 - [TBP]_{aq}$ approaches the experimental error of the individual concentrations $[TBP]_0$ and $[TBP]_{aq}$. As shown in the data tabulated below, the increasing replicate errors as D_P approach 0.1 reflected this limitation. The lowest value of D_P recorded was 0.09 ± 0.02.

$$D_P = \frac{[TBPX]_{org}}{[TBPX]_{aq}} = \frac{[TBPX]_0 - [TBPX]_{aq}}{[TBPX]_{aq}} \tag{S1}$$

Temperature Control

Temperature control of contacting experiments was achieved by heating a bead bath beneath the rotator and covering the bead bath, rotator, and headspace with a double layer of aluminum foil. The temperature was controlled by an IKA thermocouple held in the headspace to register the temperature. A separate thermometer was also held in the headspace to verify the temperature controller. Figure S6 shows that temperature at 25 °C remains satisfactorily consistent within 25.5 ± 0.5 °C over 1 h. A temperature study described below was performed to ensure that small temperature changes (1–2 °C) would not affect equilibration concentrations within the precision of this study.

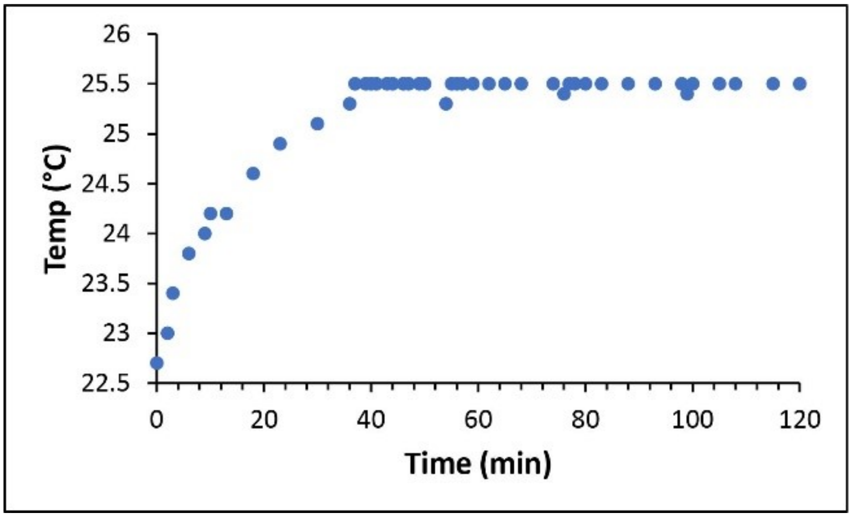

Figure S6. Temperature of headspace over 2 h using temperature control method described above.

ICP Methods

ICP-MS P calibration. 1000 ppm P ICP-MS calibration solution (H_2PO_4 in H_2O) and 1000 ppm Y ICP-MS external standard solution (Y_2O_3 in 2% v/v HNO_3) were purchased from Inorganic Ventures. P ICP-MS calibration solution was diluted with 18 MΩ deionized water to make a 1 ppm P ICP-MS calibration solution (15 mL). Further calibration solutions of 0.1, 0.01, 0.001, and 0.0001 ppm P (15 mL) were made through serial dilution with 18 MΩ deionized water starting from the 1 ppm calibration solution. Calibration solutions and a blank solution consisting of 18 MΩ deionized water were acidified up to 2% v/v HNO_3 using concentrated trace metal HNO_3. 0.1 ppm Y was added to each calibration solution and blank solution from a 5 ppm Y ICP-MS external standard solution. Calibration standard measurements which deviated by more than 70% from the Y concentration in the blank solution were discarded. Exact P and Y concentration for each calibration solution was calculated from the mass and densities of the solutions added or removed.

Representative ICP-MS P Calibration and %Y Recovery in Calibration Solutions.

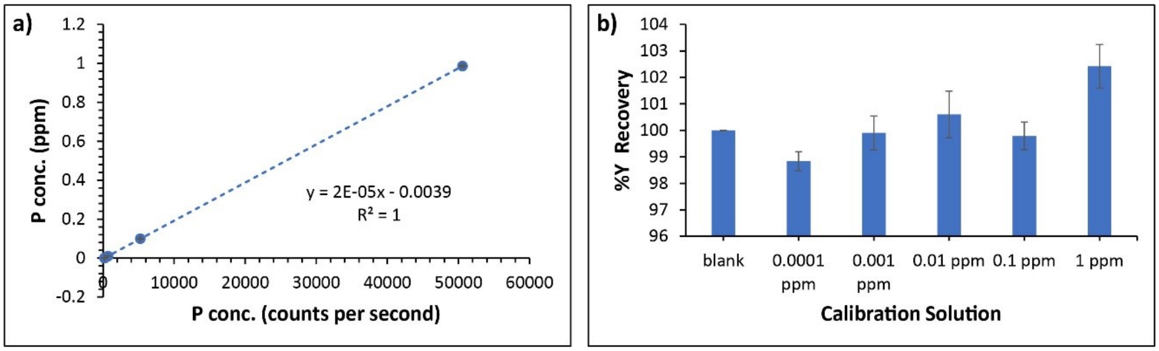

Figure S7. a) Representative P ICP-MS calibration curve. b) Representative %Y recovery (defined here as percent difference in Y concentration of the calibration solution compared to the blank solution) in each calibration solution.

ICP-MS sample preparation. 1000 ppm Y ICP-MS external standard solution (Y_2O_3 in 2% v/v HNO_3) was purchased from Inorganic Ventures. 0.2 mL of the aqueous phase of the contacting experiments were diluted with 0.8 mL 18 MΩ deionized water. Samples were acidified to 2% v/v HNO_3 with 30.8 µL concentrated trace metal HNO_3. 21.4 µL of a 5 ppm Y ICP-MS external standard solution (0.1 ppm) was added to each sample. P and Y concentrations were measured on an iCAP RQ ICP-MS by Thermo Scientific equipped with a CETAC ASX-500 autosampler. Sample measurements which deviated by more than 70% from the Y concentration in the blank solution were discarded. Extraction experiments were performed in duplicate or triplicate.

Representative ICP-MS Analysis of TBPX extraction

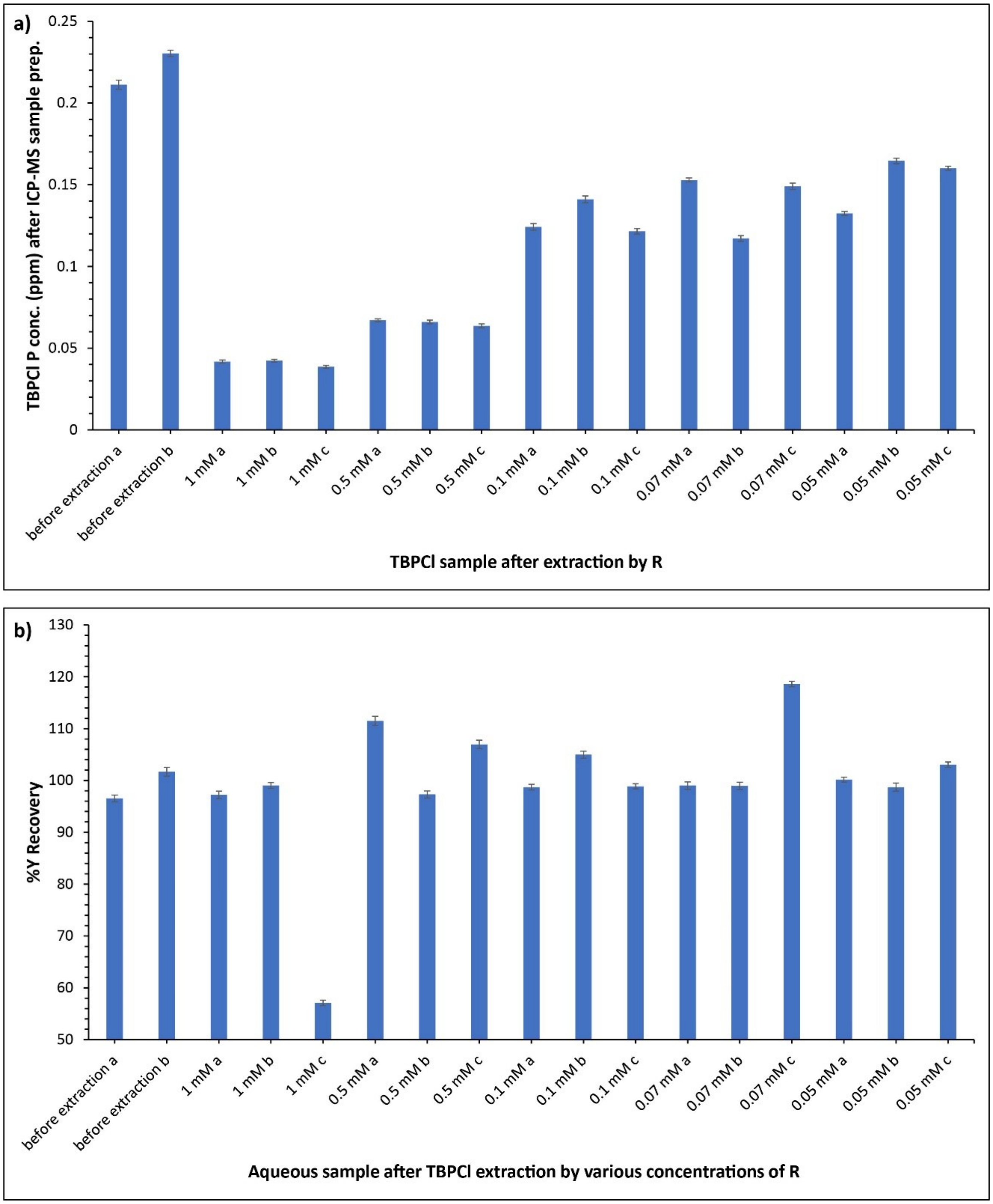

Figure S8. a) Representative TBPCl concentrations determined through ICP-MS before and after extraction with various concentrations of **R**. b) Representative %Y recovery in extraction samples. Samples that had a greater than 30% difference from 100% recovery were discarded (e.g., Sample 1 mM c).

DISTRIBUTION RESULTS

Results are shown in Tables S1–S4 for partitioning of TBPX salts into nitrobenzene without anion receptor at 25 °C.

Table S1. TBPCl partitioning from water into nitrobenzene at 25 °C, measured by ICP-MS.

$[TBPCl]_0$ (μM)	$[TBPCl]_{aq}$ (μM)	$D_{P,0}$
79.2 ± 0.8	67 ± 2	0.17 ± 0.03
60.4 ± 0.5	55.0 ± 0.1	0.101 ± 0.009
40.0 ± 0.3	36 ± 2	0.13 ± 0.04
21.1244 ± 0.0001	19.3390 ± 0.0003	0.09 ± 0.02
4.32 ± 0.08	3.6 ± 0.2	0.20 ± 0.05

Table S2. TBPBr partitioning from water into nitrobenzene at 25 °C, measured by ICP-MS.

$[TBPBr]_0$ (μM)	$[TBPBr]_{aq}$ (μM)	$D_{P,0}$
305 ± 2	210 ± 7	0.45 ± 0.04
167 ± 1	119.8 ± 0.7	0.39 ± 0.01
31.068 ± 0.004	21.952 ± 0.002	0.41 ± 0.02
0.98 ± 0.03	0.7 ± 0.2	0.4 ± 0.3

Table S3. TBPI partitioning from water into nitrobenzene at 25 °C, measured by ICP-MS.

$[TBPI]_0$ (μM)	$[TBPI]_{aq}$ (μM)	$D_{P,0}$
429 ± 3	103 ± 5	3.17 ± 0.06
213 ± 1	48.9 ± 0.5	3.36 ± 0.04
40.6 ± 0.4	10.7 ± 0.2	2.79 ± 0.05
4.87 ± 0.05	1.1 ± 0.1	3.3 ± 0.2

Table S4. $TBPNO_3$ partitioning from water into nitrobenzene at 25 °C, measured by ICP-MS.

$[TBPNO_3]_0$ (μM)	$[TBPNO_3]_{aq}$ (μM)	$D_{P,0}$
5650 ± 80	2530 ± 10	1.23 ± 0.04
527 ± 10.	248.4 ± 0.9	1.12 ± 0.04
56.7 ± 0.4	27 ± 1	1.12 ± 0.05

Results are shown in Tables S5–S8 for extraction of TBPX salts into nitrobenzene in the presence of anion receptor at 25 °C.

Table S5. TBPCl extraction from water into nitrobenzene by **R** at 25 °C, measured by ICP-MS.

$[R]_0$ (mM)	$[TBPCl]_0$ (μM)	$[TBPCl]_{aq}$ (μM)	D_P
2	32.4 ± 0.1	4.8 ± 0.2	5.80 ± 0.06
1.5	32.4 ± 0.1	5.1 ± 0.6	5.4 ± 0.2
1	25 ± 1	4.6 ± 0.2	4.4 ± 0.3
0.5	25 ± 1	7.4 ± 0.2	2.4 ± 0.2
0.1	25 ± 1	14.6 ± 1.0	0.7 ± 0.1
0.07	25 ± 1	16 ± 2	0.6 ± 0.1
0.05	25 ± 1	18 ± 2	0.4 ± 0.1

Table S6. TBPBr extraction from water into nitrobenzene by **R** at 25 °C, measured by ICP-MS.

$[R]_0$ (mM)	$[TBPBr]_0$ (μM)	$[TBPBr]_{aq}$ (μM)	D_P
2	32.0 ± 0.3	3.1 ± 0.1	9.4 ± 0.1
1.5	32.0 ± 0.3	3.5 ± 0.3	8.1 ± 0.1
1	25.12 ± 0.07	4.0 ± 0.4	5.2 ± 0.1
0.5	25.12 ± 0.07	6.2 ± 0.6	3.02 ± 0.09
0.1	25.12 ± 0.07	1.3 ± 0.4	0.96 ± 0.03
0.07	25.12 ± 0.07	1.21 ± 0.07	1.081 ± 0.009
0.05	25.12 ± 0.07	1 ± 1	0.98 ± 0.10

Table S7. TBPI extraction from water into nitrobenzene by **R** at 25 °C, measured by ICP-MS.

$[R]_0$ (mM)	$[TBPI]_0$ (μM)	$[TBPI]_{aq}$ (μM)	D_P
2	26.9 ± 0.2	3.3 ± 0.1	7.11 ± 0.08
1.5	26.9 ± 0.2	3.5 ± 0.3	6.66 ± 0.08
1	24.25 ± 0.02	3.8 ± 0.6	5.4 ± 0.2
0.5	24.25 ± 0.02	4.0 ± 0.3	5.04 ± 0.07
0.1	24.25 ± 0.02	6.2 ± 0.6	2.93 ± 0.09
0.07	24.25 ± 0.02	6.8 ± 0.7	2.54 ± 0.10
0.05	24.25 ± 0.02	6.6 ± 0.6	2.70 ± 0.09

Table S8. $TBPNO_3$ extraction from water into nitrobenzene by **R** at 25 °C, measured by ICP-MS.

$[R]_0$ (mM)	$[TBPNO_3]_0$ (μM)	$[TBPNO_3]_{aq}$ (μM)	D_P
2	36.00 ± 0.01	3.4 ± 0.2	9.54 ± 0.06
1.5	36.00 ± 0.01	3.7 ± 0.2	8.6 ± 0.1
1	37.6 ± 0.1	4.7 ± 0.2	7.00 ± 0.06
0.5	37.6 ± 0.1	6.2 ± 0.3	5.08 ± 0.05
0.1	37.6 ± 0.1	12.9 ± 0.4	1.91 ± 0.03
0.07	37.6 ± 0.1	14.4 ± 0.4	1.62 ± 0.03
0.05	37.6 ± 0.1	15.7 ± 0.4	1.40 ± 0.03

Temperature Effect on D_P. Because equilibrium constants in extraction experiments are temperature-dependent and the temperature-control method is acceptable within a range (±0.5 °C), a temperature study was performed to determine if such small changes in temperature would result in significant D_P variation. The temperature control was described above (see Figure S6). Extraction was performed with aqueous solutions of TBPCl, TBPBr, TBPI, and $TBPNO_3$ (0.04 mM) and nitrobenzene alone or 0.1 mM **R** in nitrobenzene at 30, 35, and 40 °C. D_P values determined for each of these experiments were compared with D_p results determined at 25 °C (Figures S9a-d). Results show that small variation in temperature (±0.5 °C) would be expected to give a variation of calculated D_P in the range 5–10%, which is considered tolerable but obviously a likely important contributor to experimental uncertainty in this study. While temperature control at the elevated temperatures was not precise enough to determine thermodynamic contribution to extraction, we note that D_P appears to increase with increasing temperature for all experiments with the exception of TBPI (the only lipophilic salt studied) partitioning into nitrobenzene.

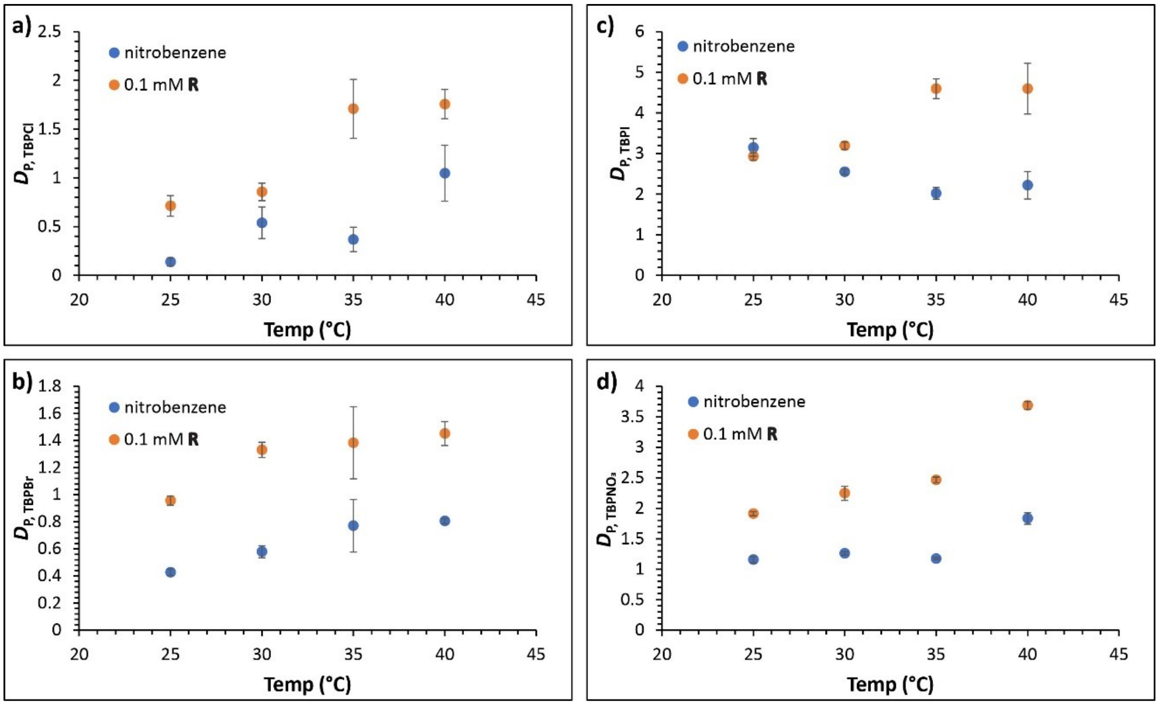

Figure S9. Temperature effect on D_P for a) TBPCl, b) TBPBr, c) TBPI, and d) $TBPNO_3$ salts with and without receptor

EQUILIBRIUM ANALYSIS OF EXTRACTION OF TBPX SALTS

Mass-Action Equations

Based on the simplified extraction model depicted in Figure 2, mass-action analysis of the obtained host-mediated LLE data yields corresponding equilibrium constants. The equations explicitly treat only the host-guest complex with 1:1 binding stoichiometry with the understanding that general complex stoichiometries ($\mathbf{R}_m X_n^-$) can be accommodated in a

straightforward, albeit more complicated, manner. Equations S2 and S3 give the equilibrium quotients for salt partitioning and organic-phase complexation, respectively, where y represents the activity coefficient of the species in solution, and an overbar denotes that species are in the organic phase. Under the expected conditions in which ions are dissociated in both phases, the host-mediated extraction constant of dissociated ions ($K_{ex\pm}$) is the product of the salt partitioning constant (K_p) and the formation constant (K_f) of anion binding with host **R** (Equation S4). Correspondingly, the Gibbs free energy of extraction of dissociated TBPX ions ($\Delta G°_{ex\pm}$) in host-mediated LLE is the sum of Gibbs free energy of partitioning ($\Delta G°_p$) of both ions TBP^+ and X^- and Gibbs free energy of formation ($\Delta G°_f$) of the host-guest complex (Equation S5). By quantifying the equilibria that occur during TBPX extraction with **R**, we will have a better understanding of the influence of the host on D_P and its ability to disrupt the Hofmeister bias.

$$K_p(TBPX) = \frac{\bar{y}_{TBP}[\overline{TBP}^+]\bar{y}_X[\bar{X}^-]}{y_{TBP}[TBP^+]y_X[X^-]} \quad \text{(S2)}$$

$$K_f(RX^-) = \frac{\bar{y}_{RX}[\overline{RX}^-]}{\bar{y}_R[\bar{R}]\bar{y}_X[\bar{X}^-]} \quad \text{(S3)}$$

$$K_{ex\pm}(TBPRX) = \frac{\bar{y}_{TBP}[\overline{TBP}^+]\bar{y}_X[\overline{RX}^-]}{y_{TBP}[TBP^+]y_X[X^-](\bar{y}_R[\bar{R}])} = K_p(TBPX) \cdot K_f(RX^-) \quad \text{(S4)}$$

$$\Delta G^{\circ}_{ex\pm} = \Delta G^{\circ}_p(TBPX) + \Delta G^{\circ}_f(RX^-) = \Delta G^{\circ}_p(TBP^+) + \Delta G^{\circ}_p(X^-) + \Delta G^{\circ}_f(RX^-) \quad \text{(S5)}$$

Salt Extraction Equilibria

Understanding salt partitioning from water into nitrobenzene is the first step before studying the effect of an anion receptor on salt extraction. $\Delta G°_p$(TBPX) can be related to K_p (defined in Equation S2) according to Equation S6. Noting that the distribution ratio of cations and the distribution ratio of anions are equal (given that TBPX is the only aqueous analyte), D_P can be substituted into Equation S6, thereby relating $\Delta G°_p(TBP^+)$ to the readily measured quantity D_P for all salts:

$$\frac{\Delta G^{\circ}_p(TBP^+) + \Delta G^{\circ}_p(X^-)}{2.303RT} = \log K_p(TBPX) = \log\left(\frac{\bar{y}_{TBP}\bar{y}_X}{y_{TBP}y_X}{D_P}^2\right) \quad \text{(S6)}$$

It may be seen that, at low electrolyte concentrations where activity coefficients approach unity, $D_P = K_p(TBPX)^{-1/2}$ and is thus independent of the TBPX concentration:

$$\log D_{P,0} = 0.5\log K'_p \quad \text{(S7)}$$

Where $D_{P,0}$ refers to the phosphorus distribution at zero receptor concentration (see Figure 3).

Liquid-liquid extractions of various concentrations of TBPX salts (0.0014–12.62 mM) were performed with nitrobenzene in the absence of the host (Tables S1–S4). ICP-MS analysis of P

concentration in the aqueous phase before and after extraction was used to calculate the distribution ratio in the absence of any receptor ($D_{P,0}$) via Equation 1. Experimental values of log $D_{P,0}$ plotted against aqueous salt concentration at equilibrium ($[TBPX]_{aq}$) for Cl^-, Br^-, I^-, and NO_3^- (Figure 3) confirm that log $D_{P,0}$ is independent of salt concentration, consistent with Equation S6. Slight activity-coefficient induced curvature may be noted at the higher TBPX concentrations.

Values for log K_p for TBPCl, TBPBr, TBPI, and $TBPNO_3$ calculated from $D_{P,0}$ in a spreadsheet according to Equation S6 are shown in Table S9. Ion activity coefficients in both phases were calculated with the Debye–Hückel equation.[3,4] The analysis was repeated with the SXLSQI software (vide infra) with the same results within experimental error; the SXLSQI results are adopted and given in the main text (Table 1). Values of log K_p for each salt shown in Table S9 were used to independently calculate $\Delta G°_p(TBP^+)$ via Equation S6 using the reported values for $\Delta G°_p(X^-)$.[5] It should be noted that use of alternative published values of $\Delta G°_p(X^-)$ gives a range of values of $\Delta G°_p(TBP^+)$ from –23 to –28 kJ/mol (Table S10, see below).

Table S9. Determination of $\Delta G°_p(TBP^+)$ using spreadsheet calculations and Debye-Hückel treatment.[a]

Salt	Average log K_p	Equilibrium	$\Delta G°_p(X^-)$ (kJ/mol)[5]	$\Delta G°_p(TBP^+)$ (kJ/mol)
**TBPCl**	–1.8 ± 0.3	$TBP^+_{(aq)} + Cl^-_{(aq)} \rightleftharpoons TBP^+_{(org)} + Cl^-_{(org)}$	35	–25 ± 1
**TBPBr**	–0.79 ± 0.04	$TBP^+_{(aq)} + Br^-_{(aq)} \rightleftharpoons TBP^+_{(org)} + Br^-_{(org)}$	29	–25 ± 1
**TBPI**	0.98 ± 0.06	$TBP^+_{(aq)} + I^-_{(aq)} \rightleftharpoons TBP^+_{(org)} + I^-_{(org)}$	18	–24 ± 1
**$TBPNO_3$**	0.06 ± 0.01	$TBP^+_{(aq)} + NO_3^-{}_{(aq)} \rightleftharpoons TBP^+_{(org)} + NO_3^-{}_{(org)}$	24	–24 ± 1
			Average =	–24 ± 1

[a]Single-ion standard Gibbs energies of partitioning shown here use the TATB (tetraphenylarsonium tetraphenylborate) extrathermodynamic assumption.[5]

The equilibrium analysis reveals the push-pull dynamics of the individual ions involved in TBPX salt partitioning as well as the previously unknown value of the standard Gibbs Energy partitioning of the TBP^+ cation, $\Delta G°_p(TBP^+)$. Although it does not have direct bearing upon determination of the extraction and binding constants for host-mediated anion extraction, it is insightful to examine the standard Gibbs energies of ion partitioning, $\Delta G°_p(TBP^+)$ and $\Delta G°_p(X^-)$. These quantities may be obtained from log K_p as converted to corresponding Gibbs energies, $\Delta G°_p(TBPX) = -2.303RT \log K_p$. Values of $\Delta G°_p(X^-)$ for anion partitioning from water into nitrobenzene at 25 °C have been well-documented,[5] as listed in Table 1. Partitioning of all the chosen anions into the organic phase is highly unfavorable, as shown by their positive values of $\Delta G°_p(X^-)$. The four values of $\Delta G°_p(TBP^+)$ derived by difference are identical (–24 ± 1 kJ/mol), revealing a compensating favorable partitioning of TBP^+ from water into nitrobenzene at 25 °C. TBP^+ is thus a highly lipophilic cation, making TBP salts excellent candidates for studying extraction with hydrophilic anions. Not surprisingly, our value of –24 kJ/mol for $\Delta G°_p(TBP^+)$ agrees with the published value –24 kJ/mol for the often-used tetra-*n*-butylammonium cation (TBA^+).[6] Judging by the difference in ionic radii between the PH_4^+ and NH_4^+ cations,[5] the molar volume of TBP^+ would be expected to be only 7 cm^3/mol larger than that of TBA^+, that is, 287

cm^3/mol vs 280 cm^3/mol.[7] For such large organic cations, the cavity term (essentially the hydrophobic effect) dominates the partition Gibbs energy, making molar volume the major determining variable.[8] Thus, one might expect that $\Delta G°_p(TBP^+)$ might be slightly more favorable than $\Delta G°_p(TBA^+)$, but the data did not indicate any difference within experimental precision.

Alternative calculations of the standard Gibbs energy of partitioning of the tetra-*n*-butylphosphonium (TBP⁺) cation between water and nitrobenzene. While a critical compilation[5] of standard Gibbs energies of partitioning of anions $\Delta G°_p(X^-)$ was used for calculating the standard Gibbs energies of partitioning of the TBP^+ cation $\Delta G°_p(TBP^+)$ with good consistency (Tables 1 and S9), alternative published values of $\Delta G°_p(X^-)$ give a range of $\Delta G°_p(TBP^+)$ from –23 kJ/mol to –28 kJ/mol, as shown in Table S10. The TATB (tetraphenylarsonium tetraphenylborate) extrathermodynamic assumption was used as the basis for these single-ion partitioning Gibbs energies.

Table S10. Determination of $\Delta G°_p(TBP^+)$ using alternative values of $\Delta G°_p(X^-)$.[a]

	log K_p (TBPX)[b]	$\Delta G°_p$ (X^-)[c]	$\Delta G°_p$ (TBP^+)	$\Delta G°_p$ (X^-)[d]	$\Delta G°_p$ (TBP^+)	$\Delta G°_p$ (X^-)[e]	$\Delta G°_p$ (TBP^+)	$\Delta G°_p$ (X^-)[f]	$\Delta G°_p$ (TBP^+)
TBPCl	–1.8	35	–24.7	29.7	–19.4	38.2	–27.9	38.8	–28.6
TBPBr	–0.79	29	–24.5	28.5	–24.0	30.5	–26.0	32.0	–27.5
TBPI	0.98	18	–23.6	18.8	–24.4	19.5	–25.1		
$TBPNO_3$	0.06	24	–24.3			26.6	–26.9	27.8	–28.1
		Avg =	–24.3 ±0.5	Avg =	–22.6 ±2.8	Avg =	–26.5 ±1.2	Avg =	–28.0 ±0.5

[a]Calculated according to $\Delta G°_p(TBP^+) + \Delta G°_p(X^-) = -RT \log K_p(TBPX)$.
[b]This work; see Table S9.
[c]From compilation.[3]
[d]From salt extraction.[6]
[e]From galvani potentials.[9]
[f]From cesium salt extraction[1] using the value 15.4 kJ/mol[6] for $\Delta G°_p(Cs^+)$.

Data Fitting Using SXLSQI

Model Parameterization. The software SXLSQI[10,11] was used to fit the extraction data in Tables S1–S8 to liquid–liquid equilibrium reactions defined in Tables 1 and 2. The program SXLSQI (Solvent eXtraction Least SQuares—Ion) is so named because ionic organic-phase species could be postulated. The treatment of nonideality is the same as described for the program SXFIT,[12] which can handle more species; SXLSQI is easier to use, handles the number of species in the present case, and was therefore adopted for the present analysis. Given the low solute concentrations in salt-extraction systems studied here, the activity corrections are expected to be small. Parameters needed by SXLSQI are shown in Tables S11–S14.

Goodness of fit is reported by SXLSQI in terms of the agreement factor σ:

$$\sigma = \sqrt{\frac{\sum_i w_i (Y_i - Y_{c,i})^2}{N_o - N_P}} \qquad \text{(S8)}$$

where Y_i is the *i*th experimentally observed quantity (i.e., D_P), $Y_{c,i}$ is the corresponding quantity calculated from the model, w_i is the weighting factor defined as the reciprocal of the square of the estimated uncertainty of Y_i , N_o is the number of observations, and N_P is the number of adjustable parameters (i.e., log *K*). Assuming experimental uncertainty for each point is properly estimated, an agreement factor of unity corresponds to a perfect fit. An agreement factor of 2, for example, may be interpreted as a fit that is on average within a factor of 2 of experimental error. Experimental errors were estimated on the basis of the standard deviation within replicated data points, which tends to underestimate actual precision over wider ranges of experimental conditions and can be affected to a greater extent by temperature, sampling, and instrumental fluctuations. Consequently, agreement factors as high as 6 were encountered, while standard relative errors of fitting were typically less than ±20%.

Table S11: Molecular weights and volume parameters for solution constituents.[a]

Constituent	Formula weight ($g\,mol^{-1}$)	V_0 ($cm^3\,mol^{-1}$)	S_v ($cm^3\,L^{1/2}\,mol^{-1/2}$)
H^+	1.008	0	0
TBP^+	259.438	288[b]	0
Cl^-	35.453	18.07	0.95
Br^-	79.904	24.79	0.611
NO_3^-	62.005	29.00	–0.812
I^-	126.904	36.24	0.250
R	753.343	520.2[c]	

[a]Unless otherwise noted, the Masson coefficients V_0 and S_v were taken from Millero.[13]
[b]Obtained by incrementing the radius of tetra-*n*-butylammonium ion by 0.32 Å, the difference between the radii of PH_4^+ and NH_4^+ cations.[5]
[c]Estimated from group contributions.[14]

Table S12. Hildebrand solubility parameters.

Species	δ ($cal^{1/2}$•$(cm^{-3})^{1/2}$ at 25 ˚C
Solvent (nitrobenzene)[a]	11.00
**R**[b]	12.6
TBP^+ (species 1)[c]	7.9
Cl^- (species 2a)[d]	8.3
Br^- (species 2b)[d]	10.2
NO_3^- (species 2c)[d]	12.6
I^- (species 2d)[d]	13.0
**R**X^- (species 3)	12.6
$\mathbf{R}_2X^-$ (species 4)	12.6

[a]Value for nitrobenzene taken from Barton.[14]
[b]Estimated from group contributions.[14]

[c]Taken as the value for tetra-*n*-butylammonium ion reported in Baes and Moyer.[15]
[d]Value taken from Baes and Moyer.[15]

Table S13. Aqueous Pitzer parameters for the interactions between cations and anions.[a]

Species	$\beta^{(0)}$	$\beta^{(1)}$	C^{Φ}
H^+–Cl^-	0.1775	0.2945	0.00080
H^+–Br^-	0.2085	0.3477	0.00152
H^+–NO_3^-	0.1168	0.3546	–0.00539
H^+–I^-	0.2211	0.4907	0.00482
TBP^+–Cl^-	0.2444[b]	–0.422[b]	–0.0639[b]
TBP^+–Br^-	–0.0347[b]	–0.499[b]	0.0002[b]
TBP^+–NO_3^-	–0.0277[c]	–0.525[c]	0.0011[c]
TBP^+–I^-	–0.3782[b]	–1.1255[b]	0.0412[b]

[a]Unless otherwise noted, values were taken from Pitzer.[16]
[b]Taken as a small extrapolation as a function of ionic radius from Pitzer parameters tabulated for tetra-*n*-alkylammonium salts.[16] Radii were calculated from molar volumes tabulated for tetra-*n*-alkylammonium salts[17] taking the value of 485 cm^{-3} mol^{-1} as the value for tetra-*n*-butylphosphonium vs 481 cm^{-3} mol^{-1} for tetra-*n*-butylammonium.
[c]Adopted the values for tetra-*n*-butylammonium bromide.[16]

Table S14. Diluent properties.

Diluent	Formula weight ($g\ mol^{-1}$)	Relative permittivity	Molar volume ($cm^3\ mol^{-1}$)
Nitrobenzene	11.00	34.82[a]	520.2[b]

[a]Taken from reference.[17]
[b]Estimated from group contributions.[14]

Analysis of TBP Salt Partitioning. Table S15 shows the SXLSQI analysis of the extraction of TBPX by nitrobenzene used alone, and the results are plotted in Figure S10. Good agreement may be seen compared with the analysis presented in Table S9. Standard relative errors of fitting were as follows: ±32% (Cl^-), ±7% (Br^-), ±8%(I^-), and ±1% (NO_3^-), showing excellent fits for Br^-, I^-, and NO_3^-. The chloride data were less precise because of the small difference between the initial and equilibrium concentrations of TBP^+ in this case.

Table S15. $\Delta G°_p(TBP^+)$ and log K_p calculated using SXLSQI from the data in Tables S1–S4 for TBPCl, TBPBr, TBPI, and $TBPNO_3$ partitioning from water into nitrobenzene at 25 °C.[a]

Salt	Average log K_p and σ[b]	Equilibrium	$\Delta G°_p(X^-)$ ($kJ\ mol^{-1}$)[34]	$\Delta G°_p(TBP^+)$ ($kJ\ mol^{-1}$)
**TBPCl**	–1.94 ± 0.09<br>σ = 1.53	$TBP^+_{(aq)} + Cl^-_{(aq)} \rightleftharpoons TBP^+_{(org)} + Cl^-_{(org)}$	35	–24 ± 1
**TBPBr**	–0.81 ± 0.02<br>σ = 0.96	$TBP^+_{(aq)} + Br^-_{(aq)} \rightleftharpoons TBP^+_{(org)} + Br^-_{(org)}$	29	–24 ± 1
**TBPI**	0.97 ± 0.03<br>σ = 4.59	$TBP^+_{(aq)} + I^-_{(aq)} \rightleftharpoons TBP^+_{(org)} + I^-_{(org)}$	18	–24 ± 1

**$TBPNO_3$**	0.070 ± 0.007<br>σ = 3.58	$TBP^+_{(aq)} + NO_3^-{}_{(aq)} \rightleftharpoons TBP^+_{(org)} + NO_3^-{}_{(org)}$	24	−24 ± 1
			Average =	−24 ± 1

[a]Single-ion standard Gibbs energies of partitioning shown here use the TATB (tetraphenylarsonium tetraphenylborate) extrathermodynamic assumption.[5]
[b]Agreement factor (eq. S7) obtained in fitting the data.

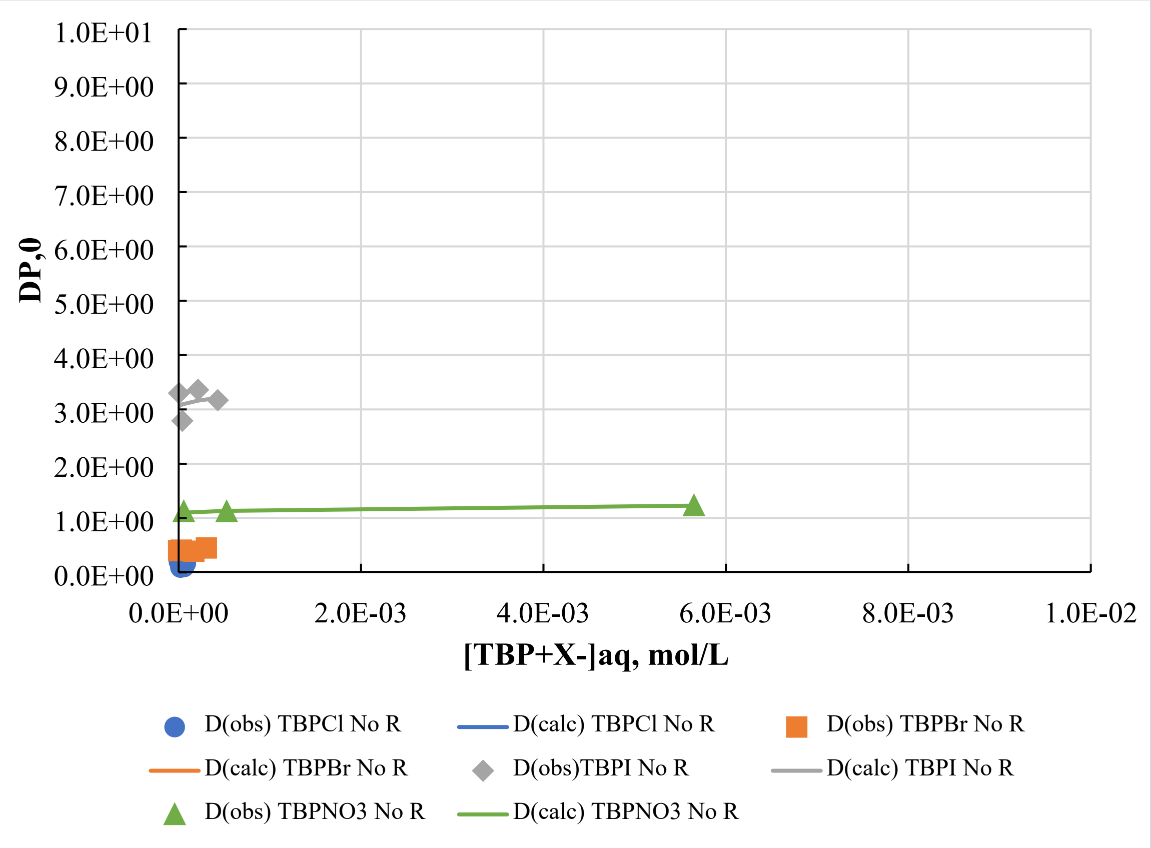

Figure S10. Calculated vs observed values of $D_{P,0}$. Observed values were taken from Tables S1–S4. Calculated values were output from SXLSQI using the log K_p values shown in Table 1.

Host-Mediated TBPX Salt Extraction. Figure S11 shows the host-mediated extraction of TBPX salts into nitrobenzene solution at 25 °C. Values of log D_P are plotted as a function of log([**R**]$_0$/[TBPX]$_0$) for a) TBPCl, b) TBPBr, c) TBPI, and d) $TBPNO_3$ in comparison with the values of log D_P for TBPX partitioning in the absence of host, shown as the horizontal lines. As the host becomes progressively more dilute, the points descend asymptotically toward these horizontal baselines, each shown with their margins of uncertainty. Corresponding plots of log D_P vs log [**R**]$_0$ are given in the main text (Figure 4).

While the extraction of all four salts increases with addition of host **R**, more important is the difference (i.e., enhancement) between these high D_P values and the baseline $D_{P,0}$ for background TBPX extraction **at zero receptor concentration**. Accordingly, the presence of an

anion receptor increases log D_P compared to log $D_{P,0}$ for TBPCl the most and TBPI the least, as one would expect for stronger binding of the smaller anion. Qualitatively, the enhancement of extraction of TBPX by R follows a reverse (vs attenuated) Hofmeister bias: $Cl^- > Br^- > NO_3^- > I^-$.

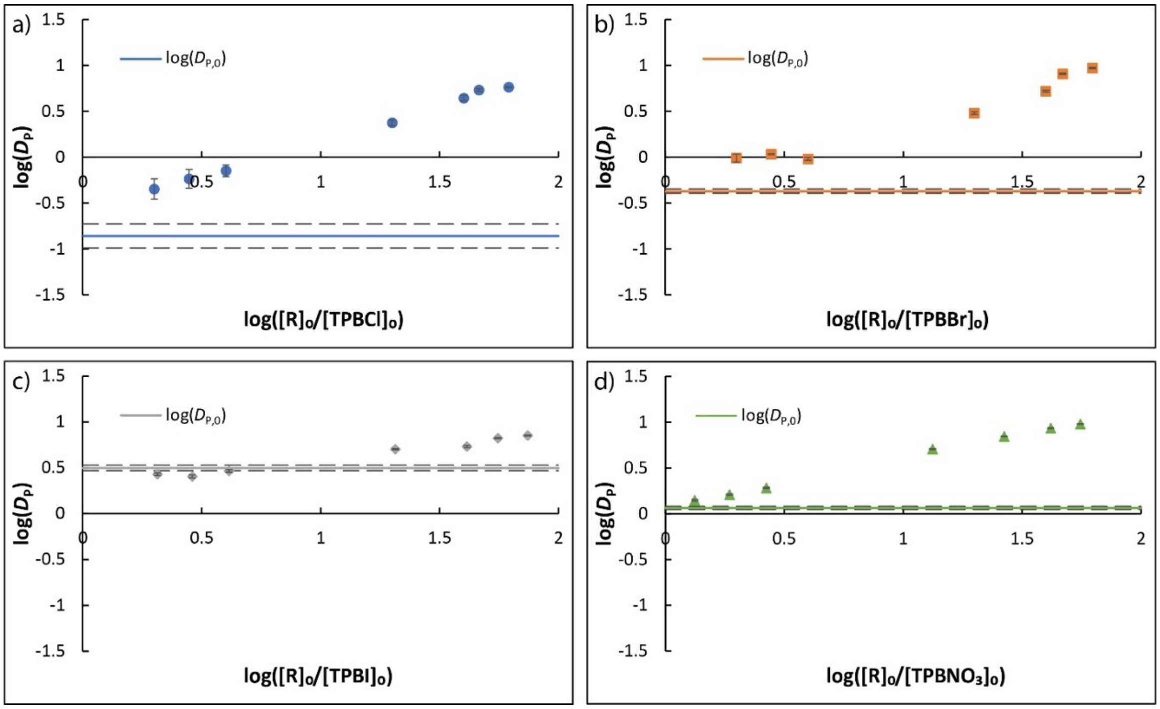

Figure S11. Experimentally determined log D_P plotted as a function of log([**R**]$_0$/[TBPX]$_0$) for a) TBPCl, b) TBPBr, c) TBPI, and d) $TBPNO_3$. Solid lines demark $D_{P,0}$ for each salt, and dashed lines demark the standard replicate error.

Slope analysis of host-mediated extraction of TBPX. Qualitative understanding of the binding stoichiometry may be obtained from simplified linear analysis. Mass-action equations quantitatively considering the background extraction of TBPX salts, loading, formation of higher stoichiometries, and activity coefficients rigorously produce complex nonlinear expressions that do not lend themselves readily to graphical or simple spread-sheet analysis. However, if the background extraction of TBPX can be neglected, expanding Equation S4 as a log–log expression and noting that [TBP^+] = [X^-], gives the very simple Equation S9, where $K'_{ex\pm}$ represents the conditional constant equal to $K_{ex\pm}$ multiplied by the reciprocal of the quotient of activity coefficients shown in Equation S4.

$$\log D_p = 0.5\log[\overline{R}] + 0.5\log K'_{ex\pm} \quad \text{(S9)}$$

Or more generally for any number m of host molecules binding the anion:

$$\log D_p = 0.5\, m\log[\overline{R}] + 0.5\log K'_{ex\pm} \quad \text{(S10)}$$

Following Equation S10, a graphical analysis would be meaningful regarding the stoichiometry of the $\mathbf{R}_mX^-$ complex with the following assumptions: the background extraction of TBPX by nitrobenzene is small with respect to the host-mediated extraction, the extracted loading of the host is low so that $[\mathbf{R}]_{org} \approx [\mathbf{R}]_{org,0}$, and activity coefficients (or their ratios) are either constant or close to unity. For the highest four data points plotted for each anion (i.e., where $[\mathbf{R}]/[TBPX]_0 > 1$) except iodide in Figure 4, these assumptions are reasonably met, giving the following slopes in plotting log D_P vs log[R]: 0.65 ± 0.10 (Cl^-), 0.85 ± 0.04 (Br^-), and 0.46 ± 0.01 (NO_3^-). Equation S10 implies an expected slope of 0.5 if the 1:1 organic-phase complex $\mathbf{R}X^-$ is predominantly formed, which is approximately met with nitrate. The higher nonintegral slopes for Cl^- and Br^- imply likely additional formation of $\mathbf{R}_2X^-$ complexes. Although the larger size and lower charge density of Br^- would lead one to expect less tendency to add a second receptor molecule, the larger size would be spatially more accommodating, perhaps explaining its higher slope vs Cl^-.

SXLSQI Analysis of Host-Mediated TBPX Extraction. Figures S12–S15 show the fitting in the SXLSQI analysis of the host-mediated extraction of TBPX. Observed values were taken from Tables S5–S8. For chloride and bromide, a 3-species model postulating only the 1:1 complex was tested and compared with a 4-species model postulating formation of both 1:1 and 2:1 complexes; the 4-species model was adopted based on fitting statistics. For nitrate and iodide, the 3-species model was adequate to explain the data. Obtained values of log $K_{ex\pm}$ are shown in Table 2. Agreement factors and standard relative errors of fitting were as follows: 2.44, ±27% (Cl^-, 4-species model); 3.38, ±64% (Cl^-, 3-species model); 5.82, ±16% (Br^-, 4-species model); 16.9, ±32% (Br^-, 3-species model); 5.67, ±17% (I^-, 3-species model); and 7.69, ±13% (NO_3^-, 3-species model). Standard errors of fitting reflect the scatter about the calculated lines.

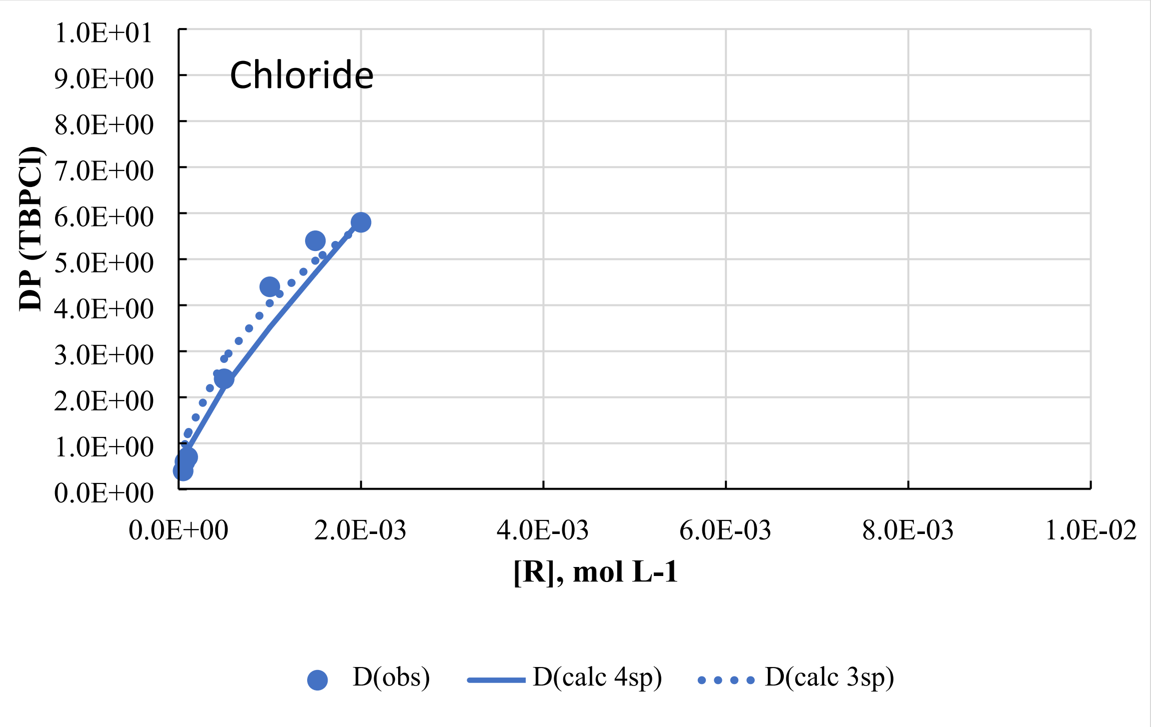

Figure S12. Calculated vs observed values of D_P for extraction of TBPCl as a function of [**R**].

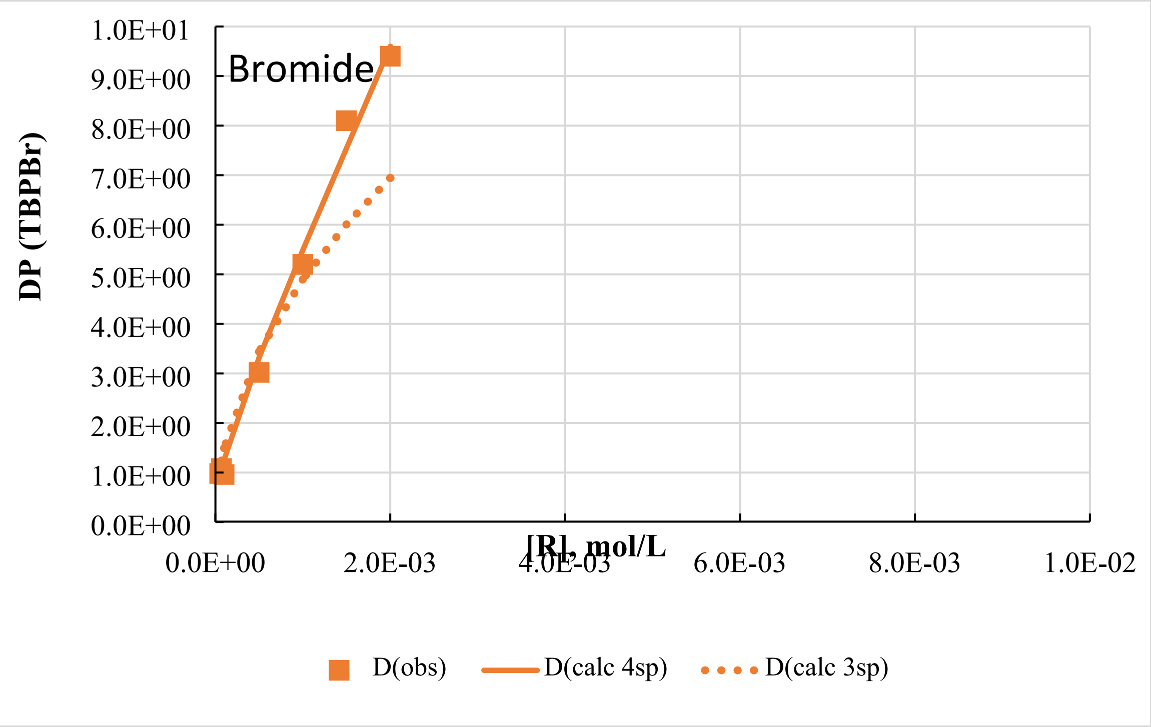

Figure S13. Calculated vs observed values of D_P for extraction of TBPBr as a function of [**R**].

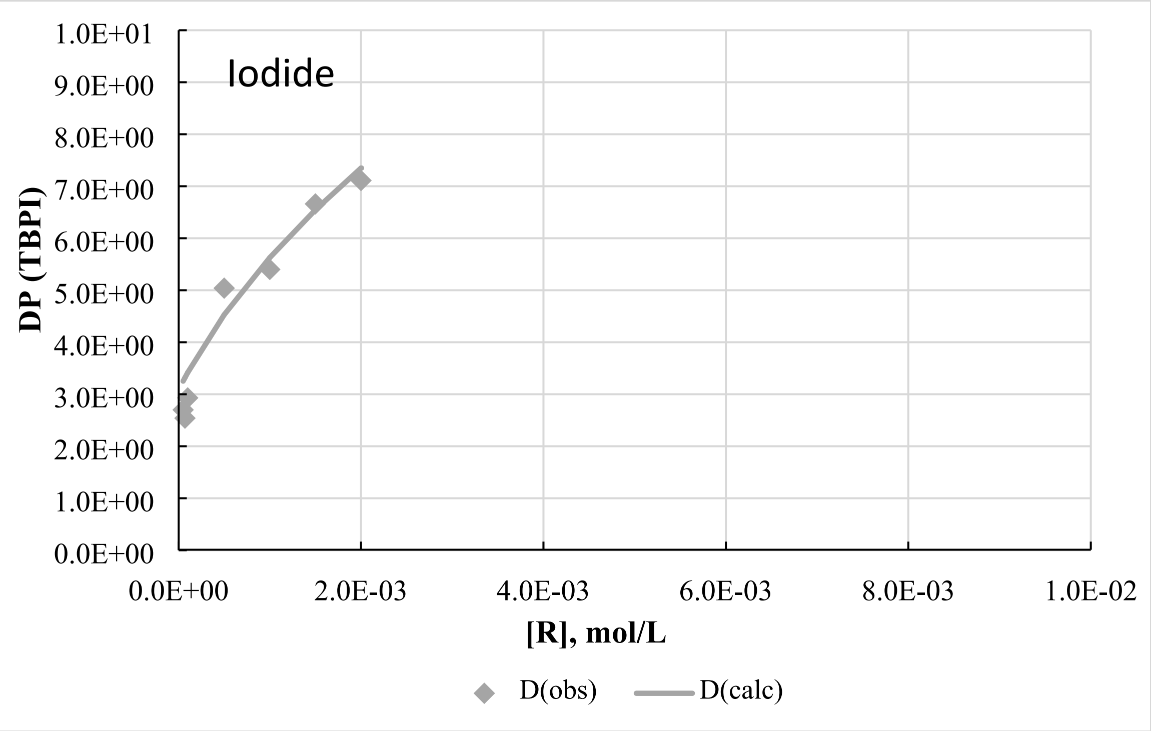

Figure S14. Calculated vs observed values of D_P for extraction of TBPI as a function of [**R**].

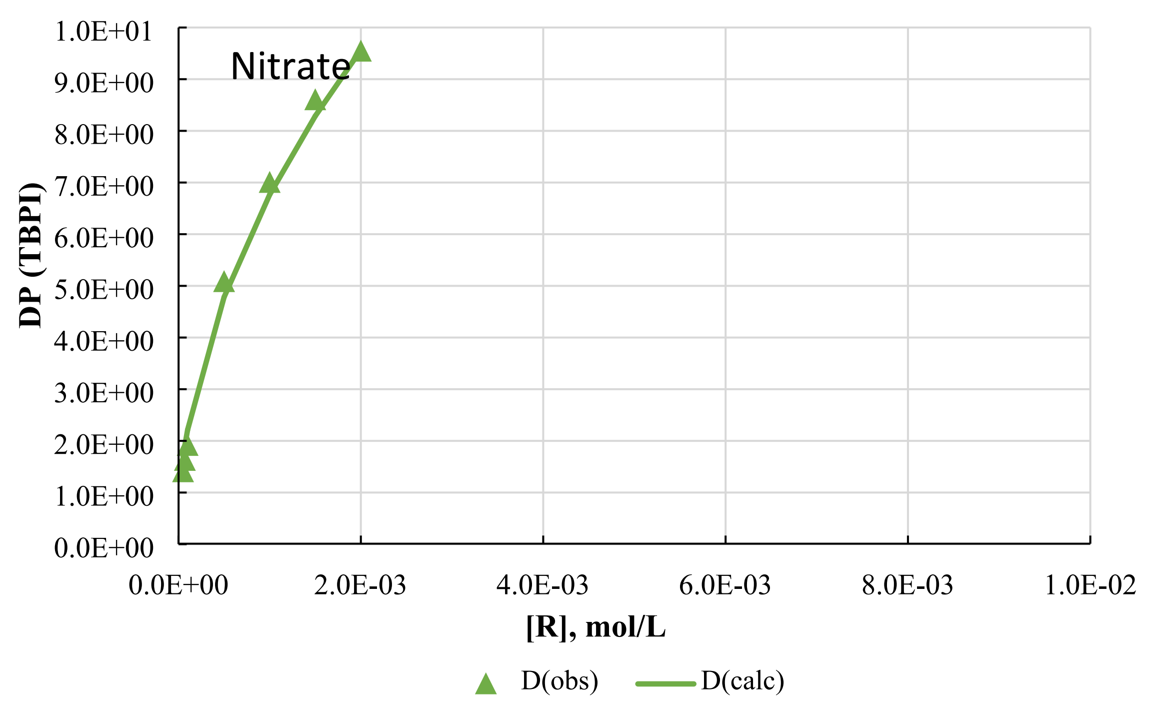

Figure S15. Calculated vs observed values of D_P for extraction of $TBPNO_3$ as a function of [**R**].

COMPUTATIONAL METHODS AND ANALYSIS

Density functional theory (DFT) calculations were carried out using the Gaussian 16 Revision C.02[18] software packages. The structures of free ligands and anion complexes were optimized using the M06-2X flavor of density functionals[19] using the 6-31G(d) basis set. Zero-point energies and thermal corrections were obtained at the same level of theory. The standard Gibbs free energy of every species in the gas phase was calculated using the rigid rotor-harmonic oscillator approximation without scaling, except that vibrational frequencies lower than 60 cm^{-1} were raised to 60 cm^{-1}. This procedure is based on the so-called quasiharmonic approximation, which was first introduced by Truhlar et al.[20] and serves as a way to correct for the well-known breakdown of the harmonic oscillator model for the free energies of low-frequency vibrational modes. Solvent effects were described by including implicit solvent corrections for nitrobenzene using the IEFPCM[21,22] solvation model in Gaussian 16. Geometry optimization in the solvent reaction field was carried out at the M06-2X/6-31G(d) level.

The structures of both the free ligand and the 1:1 complex were generated from crystal structures of similar receptors.[23,24] Subsequently, a thorough conformational analysis was performed, which involved assessing all possible structures that could be formed by rotating around single bonds. The most stable conformations of the free ligand and the 1:1 complex with Br^- are shown in Figure S16. The structures of the 2:1 complexes were constructed using two different methods. In the first method, the ligands from the 1:1 complex were positioned in an

approximately parallel orientation, and a single halide was placed at the central point between two halides. Subsequently, one ligand was incrementally rotated to generate at least six preliminary structures for geometry optimization. In the second method, we expanded the clefts of the ligands in the 1:1 complex. This adjustment allowed a pair of ligands to simultaneously interact with a single halide anion without significant steric clashes. A range of starting geometries was generated with the planes of the two ligands varying from nearly parallel to a perpendicular orientation. It should be noted that none of the initial structures formed a helical shape, nor were the ligands manipulated to assume a helical configuration. The presence of a single halide anion induced the formation of single and double helices, aided by the rotation of terminal aromatic rings towards a more stable conformation.

To assess the feasibility of forming a 2:1 complex, we carried out calculations to determine the change in Gibbs free energy for $\mathbf{R} + \mathbf{R}X^- \rightleftharpoons \mathbf{R}_2X^-$ ($X = Cl^-$ and Br^-). The free energies associated with the two conformations, determined in the gas phase and using the implicit solvent, are given in Table S16. Table S17 presents a comparison of hydrogen bond lengths to halides between the 2:1 and 1:1 complexes.

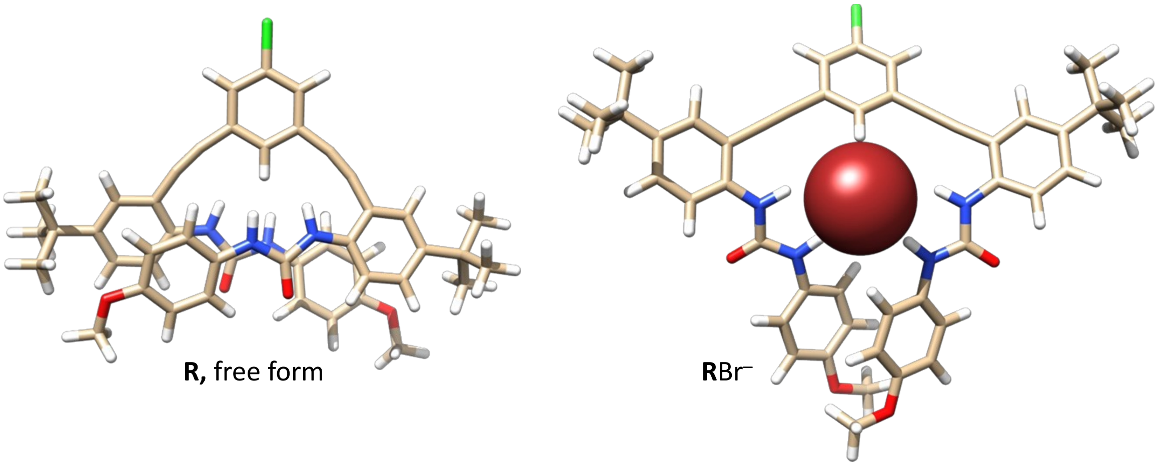

Figure S16. Optimized molecular structures of a free ligand R (left) and 1:1 complex with Br^- (right) using DFT and implicit solvent model.

Table S16. Gibbs free energies for $\mathbf{R} + \mathbf{R}X^- \rightleftharpoons \mathbf{R}_2X^-$ ($X = Cl^-$ and Br^-) computed in the gas phase and with inclusion of an implicit solvent (kcal/mol).

Type	Anion	Gas Phase	Implicit Solvent (nitrobenzene)
**Single helix**	Cl^-	−33.4	−21.3
	Br^-	−35.8	−22.1
**Double helix**	Cl^-	−33.2	−21.4
	Br^-	−3.7	−20.9

Table S17. Hydrogen bond distances from urea groups of **R** to halides in the 1:1 and 2:1 complexes obtained from DFT in the gas phase and with inclusion of an implicit solvent (angstroms).

Hydrogen bond	1:1 Complex	Single helix	Double helix
Gas phase			
$N_1H\cdots Cl^-$	2.14	2.21	2.24
	2.86	2.55	2.98
$N_2H\cdots Cl^-$	2.16	2.46	2.24
	2.76	3.57	2.98
$N_1H\cdots Br^-$	2.28	2.36	2.37
	2.93	2.78	2.98
$N_2H\cdots Br^-$	2.33	2.53	2.37
	2.78	3.42	2.98
Implicit solvent (nitrobenzene)			
$N_1H\cdots Cl^-$	2.20	2.25	2.23
	2.85	2.58	2.94
$N_2H\cdots Cl^-$	2.26	2.38	2.23
	2.66	3.44	2.94
$N_1H\cdots Br^-$	2.34	2.37	2.38
	2.92	2.79	3.10
$N_2H\cdots Br^-$	2.42	2.49	2.41
	2.74	3.32	2.92

Table S18. Optimized Cartesian coordinates (in angstroms) and total energies (in hartrees) from calculations at the M06-2X/6-31G(d) level of theory with solvent effects included using the IEFPCM model for nitrobenzene

Free ligand **R**, E(RM062X) = –2759.07807850
H 3.2340 –4.3256 –1.1137
C 5.7388 –4.3031 –1.4295
H 6.8100 –4.4683 –1.5461
H 5.1905 –5.0823 –1.9715
H 5.4740 –4.3500 –0.3659
O 5.4769 –3.0203 –1.9601
C 4.2128 –2.5336 –1.8209
C 4.0354 –1.1858 –2.1461
C 3.1221 –3.2814 –1.3815
H 4.8947 –0.6053 –2.4663
C 2.7850 –0.6039 –2.0349
C 1.8639 –2.6903 –1.2583
C 1.6845 –1.3491 –1.5893
H 2.6613 0.4497 –2.2752

H	1.0291	−3.2688	−0.8906
N	0.4568	−0.6673	−1.4442
C	−0.8055	−1.2230	−1.4076
O	−1.0313	−2.4224	−1.4590
N	−1.7800	−0.2496	−1.3008
C	−3.1594	−0.4026	−1.1513
C	−3.8465	−1.6031	−1.3527
C	−3.9066	0.7498	−0.8073
H	−3.2907	−2.4938	−1.6093
C	−5.2285	−1.6394	−1.2207
C	−3.1811	1.9584	−0.5710
C	−5.3000	0.6794	−0.6807
H	−5.7274	−2.5879	−1.4023
C	−5.9919	−0.5105	−0.8859
C	−2.3902	2.8672	−0.4159
H	−5.8233	1.5904	−0.4131
C	−7.5168	−0.6072	−0.8082
C	−1.2717	3.7451	−0.2457
C	−8.1458	0.6874	−0.2838
C	−8.0674	−0.8831	−2.2180
C	−7.9263	−1.7558	0.1276
C	−1.3518	5.1430	−0.2761
C	−0.0250	3.1362	−0.0766
H	−7.9480	1.5336	−0.9500
H	−9.2315	0.5642	−0.2175
H	−7.7720	0.9375	0.7152
H	−7.6676	−1.8176	−2.6249
H	−7.8020	−0.0715	−2.9039
H	−9.1598	−0.9660	−2.1880
H	−7.5701	−2.7231	−0.2415
H	−9.0187	−1.8082	0.1954
H	−7.5144	−1.6068	1.1302
H	−2.3045	5.6412	−0.4102
C	−0.1794	5.8799	−0.1398
H	0.0377	2.0522	−0.0505
C	1.1455	3.8896	0.0602
C	1.0687	5.2868	0.0268
C	2.3485	3.1379	0.2633
H	1.9590	5.8951	0.1331
C	3.1908	2.2808	0.4413
C	3.9449	1.0975	0.7167
C	3.2234	−0.0234	1.1960
C	5.3234	1.0062	0.4853
N	1.8624	0.1572	1.4582
C	3.9137	−1.2202	1.4016
H	5.8286	1.8940	0.1220
C	6.0218	−0.1782	0.7032
C	0.8823	−0.8026	1.6250

H 3.3752 −2.0915 1.7462
C 5.2789 −1.2802 1.1538
C 7.5358 −0.2961 0.5164
N −0.3665 −0.2345 1.7675
O 1.0961 −2.0044 1.6341
H 5.7826 −2.2265 1.3334
C 8.1873 −0.4966 1.8958
C 8.1318 0.9599 −0.1277
C 7.8705 −1.5005 −0.3780
C −1.6017 −0.9064 1.8920
H 7.9756 0.3539 2.5524
H 7.8131 −1.4039 2.3813
H 9.2742 −0.5902 1.7912
H 7.9904 1.8455 0.5008
H 9.2085 0.8214 −0.2682
H 7.6851 1.1557 −1.1088
H 8.9555 −1.5659 −0.5173
H 7.3927 −1.4099 −1.3578
H 7.5347 −2.4425 0.0674
C −2.7162 −0.1340 2.2470
C −1.7779 −2.2645 1.6287
H −2.5962 0.9301 2.4362
C −3.9756 −0.7025 2.3308
C −3.0461 −2.8386 1.7233
H −0.9347 −2.8725 1.3366
H −4.8422 −0.1007 2.5854
C −4.1519 −2.0633 2.0665
H −3.1518 −3.8945 1.5040
O −5.4251 −2.5361 2.1707
C −5.6491 −3.8731 1.7725
H −6.7216 −4.0441 1.8655
H −5.3419 −4.0295 0.7311
H −5.1104 −4.5770 2.4174
H −1.4651 0.7059 −1.1910
H 0.5171 0.3300 −1.6042
H 1.5628 1.1225 1.4104
H −0.4117 0.7617 1.9411
Cl −0.2768 7.6203 −0.1799

RBr^-, E(RM062X) = −5330.75239390
Br −0.0994 −0.1058 −1.3441
O −0.9553 6.9358 2.2342
O 4.6252 2.2231 −0.4128
N −2.3162 1.9072 0.2616
H −1.5267 1.3355 −0.0412
N 3.6148 0.1702 −0.6609
H 2.7194 −0.3129 −0.6195
C 0.0016 −3.1761 0.6093

H	−0.0179	−2.2606	0.0278
C	1.2255	−3.7292	0.9935
C	−2.0132	3.1716	0.8108
C	−2.8399	4.2871	0.6311
H	−3.7735	4.1811	0.0944
C	−2.4621	5.5186	1.1438
H	−3.0952	6.3910	1.0130
C	−1.2463	5.6721	1.8191
C	−0.4227	4.5624	2.0101
H	0.5329	4.6510	2.5135
C	−0.8150	3.3233	1.5083
H	−0.1642	2.4624	1.6402
C	0.2721	7.1271	2.9115
H	0.3299	8.1912	3.1400
H	1.1183	6.8420	2.2783
H	0.3014	6.5512	3.8440
C	2.4379	−3.0795	0.5962
C	3.4523	−2.5300	0.2222
C	4.6862	−1.9624	−0.2244
C	4.7680	−0.6226	−0.6536
C	6.0111	−0.1514	−1.0999
H	6.0952	0.8677	−1.4479
C	7.1232	−0.9787	−1.0925
H	8.0641	−0.5674	−1.4481
C	7.0677	−2.3132	−0.6605
C	5.8302	−2.7810	−0.2377
H	5.7081	−3.8032	0.1017
C	8.3279	−3.1791	−0.6793
C	9.4012	−2.5353	0.2145
H	10.3108	−3.1459	0.2076
H	9.0481	−2.4556	1.2480
H	9.6666	−1.5320	−0.1330
C	8.8573	−3.2765	−2.1201
H	8.1110	−3.7356	−2.7769
H	9.7640	−3.8912	−2.1461
H	9.1075	−2.2912	−2.5255
C	8.0583	−4.5972	−0.1665
H	7.6999	−4.5899	0.8684
C	3.6074	1.5557	−0.5563
C	−3.5737	1.3804	0.1557
C	−1.1979	−3.8049	0.9537
C	1.2542	−4.9197	1.7351
H	8.9860	−5.1775	−0.1955
H	7.3188	−5.1162	−0.7857
N	2.3473	2.0798	−0.6257
O	−4.5979	1.9732	0.4747
N	−3.5720	0.0909	−0.3558
C	−1.1780	−4.9961	1.6933

C −2.4316 −3.2158 0.5283
H 2.1972 −5.3590 2.0393
C 0.0500 −5.5266 2.0692
C 2.0232 3.4511 −0.5921
C −4.7260 −0.6851 −0.5189
H −2.1013 −5.4938 1.9662
C −3.4522 −2.6876 0.1416
C 2.7930 4.4055 0.0855
C 0.8442 3.8564 −1.2206
C −4.6660 −2.0751 −0.3009
C −5.9450 −0.1317 −0.9293
C 2.3762 5.7280 0.1223
H 3.7099 4.1090 0.5761
C 0.4154 5.1805 −1.1694
H 0.2370 3.1133 −1.7330
C −5.8080 −2.8710 −0.4989
C −7.0604 −0.9368 −1.1006
H −6.0060 0.9334 −1.1069
H 2.9656 6.4773 0.6425
C 1.1808 6.1248 −0.4847
H −0.5240 5.4497 −1.6380
C −7.0240 −2.3253 −0.8927
H −5.7069 −3.9357 −0.3213
H −7.9863 −0.4652 −1.4188
O 0.8460 7.4373 −0.3364
C −8.2875 −3.1625 −1.0987
C −0.3827 7.8584 −0.8971
C −8.0429 −4.6495 −0.8250
C −8.7705 −3.0087 −2.5508
C −9.3854 −2.6665 −0.1424
H −0.4831 8.9149 −0.6491
H −1.2217 7.3002 −0.4685
H −0.3813 7.7386 −1.9870
H −7.7197 −4.8214 0.2072
H −8.9725 −5.2057 −0.9822
H −7.2857 −5.0643 −1.4988
H −8.0058 −3.3574 −3.2528
H −9.6785 −3.6012 −2.7090
H −9.0035 −1.9666 −2.7904
H −9.6285 −1.6143 −0.3196
H −10.3002 −3.2524 −0.2846
H −9.0672 −2.7721 0.9000
H −2.6746 −0.3823 −0.4464
H 1.5808 1.4510 −0.8741
Cl 0.0803 −7.0087 2.9925

$\mathbf{R_2}Br^-$ single helix, E(RM062X) = −8089.90073809
Br 0.0002 0.2963 0.0001

O	5.1681	–0.4275	1.1409
N	3.4596	1.1131	1.0920
H	2.4592	1.2342	0.9387
N	3.0914	–1.0343	0.3805
H	2.1445	–0.6906	0.2337
C	4.0005	–0.1508	0.8838
C	3.2320	–2.4281	0.2623
C	2.0855	–3.1407	–0.0965
H	1.1535	–2.6008	–0.2570
C	2.1154	–4.5207	–0.2715
H	1.2052	–5.0339	–0.5608
C	3.3099	–5.2131	–0.0650
C	4.4605	–4.5026	0.2857
H	5.3873	–5.0511	0.4256
C	4.4349	–3.1239	0.4378
H	5.3401	–2.5833	0.6748
C	4.1982	2.2872	1.2679
C	3.5173	3.4670	1.6357
C	4.2222	4.6712	1.8011
H	3.6442	5.5460	2.0770
C	5.5946	4.7575	1.6010
C	6.2467	3.5790	1.2081
H	7.3165	3.5945	1.0166
C	5.5801	2.3729	1.0452
H	6.1243	1.4903	0.7426
C	2.0924	3.4986	1.7526
C	0.8849	3.6076	1.8009
C	–0.5372	3.7670	1.7679
C	–1.0956	5.0076	1.4267
H	–0.4572	5.8647	1.2437
C	–2.4753	5.1150	1.3131
C	–3.3239	4.0315	1.5048
H	–4.3945	4.1339	1.3566
C	–2.7638	2.7977	1.8663
C	–1.3765	2.6788	2.0163
H	–0.9422	1.7268	2.2930
O	1.4839	–3.2967	–3.8318
N	2.4007	–1.4557	–2.7926
H	2.1685	–0.5689	–2.3568
N	0.1435	–1.7598	–2.7829
H	0.1334	–0.9770	–2.1330
C	1.3497	–2.2660	–3.1840
C	–1.1253	–2.2765	–3.1328
C	–2.1578	–1.3710	–3.3570
H	–1.9479	–0.3042	–3.3218
C	–3.4608	–1.8090	–3.5855
H	–4.2495	–1.0776	–3.7130
C	–3.7298	–3.1774	–3.5995

C	−2.6837	−4.0918	−3.4263
H	−2.9156	−5.1520	−3.4387
C	−1.3941	−3.6498	−3.1822
H	−0.5935	−4.3579	−3.0050
C	3.7570	−1.7786	−2.7572
C	4.6587	−0.7506	−2.4004
C	6.0186	−1.0379	−2.2183
H	6.6621	−0.2215	−1.9108
C	6.5260	−2.3180	−2.4136
C	5.6199	−3.3059	−2.8281
H	5.9742	−4.3192	−2.9993
C	4.2652	−3.0589	−2.9961
H	3.5904	−3.8538	−3.2800
C	7.9936	−2.6763	−2.1766
C	4.1400	0.5662	−2.2005
C	3.5623	1.6246	−2.0550
C	2.7635	2.7977	−1.8664
C	3.3236	4.0316	−1.5048
H	4.3942	4.1340	−1.3566
C	2.4750	5.1151	−1.3132
C	1.0953	5.0076	−1.4268
H	0.4568	5.8648	−1.2439
C	0.5369	3.7671	−1.7680
C	1.3762	2.6788	−2.0163
H	0.9420	1.7268	−2.2931
C	6.3865	6.0548	1.7681
C	−0.8852	3.6076	−1.8011
C	−2.0927	3.4986	−1.7528
C	−3.5176	3.4668	−1.6357
C	−4.1983	2.2870	−1.2678
C	−4.2226	4.6709	−1.8011
C	−5.5802	2.3725	−1.0449
C	−5.5951	4.7571	−1.6008
C	−6.2469	3.5785	−1.2078
H	−3.6447	5.5458	−2.0771
H	−6.1242	1.4898	−0.7423
H	−7.3168	3.5939	−1.0162
N	−3.4596	1.1129	−1.0920
H	−2.4592	1.2342	−0.9388
C	−4.0003	−0.1510	−0.8838
N	−3.0910	−1.0345	−0.3806
H	−2.1442	−0.6906	−0.2337
O	−5.1678	−0.4279	−1.1408
C	−3.2316	−2.4282	−0.2623
C	−2.0850	−3.1407	0.0967
C	−4.4343	−3.1242	−0.4378
C	−2.1148	−4.5207	0.2718
C	−4.4599	−4.5028	−0.2855

C	−3.3092	−5.2132	0.0653
H	−1.1531	−2.6007	0.2572
H	−5.3396	−2.5836	−0.6750
H	−1.2046	−5.0338	0.5612
H	−5.3866	−5.0515	−0.4254
Cl	−3.1605	6.6623	0.8807
Cl	3.1600	6.6624	−0.8808
C	−3.5625	1.6244	2.0550
C	−4.1402	0.5661	2.2004
C	−4.6588	−0.7508	2.4003
C	−6.0186	−1.0383	2.2180
C	−3.7570	−1.7787	2.7572
C	−6.5259	−2.3184	2.4132
C	−4.2651	−3.0591	2.9959
C	−5.6197	−3.3062	2.8278
H	−6.6621	−0.2219	1.9104
H	−3.5903	−3.8540	3.2799
H	−5.9740	−4.3196	2.9990
N	−2.4007	−1.4557	2.7927
H	−2.1685	−0.5689	2.3569
C	−1.3497	−2.2660	3.1841
N	−0.1435	−1.7598	2.7830
H	−0.1335	−0.9769	2.1331
O	−1.4838	−3.2967	3.8319
C	1.1253	−2.2765	3.1329
C	2.1578	−1.3710	3.3570
C	1.3941	−3.6497	3.1823
C	3.4609	−1.8090	3.5854
C	2.6837	−4.0918	3.4263
C	3.7298	−3.1773	3.5994
H	1.9479	−0.3042	3.3218
H	0.5935	−4.3578	3.0051
H	4.2495	−1.0776	3.7129
H	2.9156	−5.1520	3.4386
C	−7.9934	−2.6769	2.1761
C	−6.3871	6.0543	−1.7679
C	−8.8074	−1.4706	1.6976
H	−9.8457	−1.7737	1.5285
H	−8.8096	−0.6649	2.4395
H	−8.4154	−1.0717	0.7553
C	−8.6148	−3.1997	3.4816
H	−8.0910	−4.0885	3.8474
H	−8.5739	−2.4343	4.2636
H	−9.6641	−3.4698	3.3182
C	−8.0772	−3.7721	1.0988
H	−7.5382	−4.6757	1.4005
H	−9.1233	−4.0456	0.9198
H	−7.6468	−3.4199	0.1545

C	8.8076	−1.4699	−1.6984
H	8.4156	−1.0709	−0.7561
H	9.8459	−1.7729	−1.5294
H	8.8095	−0.6643	−2.4404
C	8.0777	−3.7714	−1.0993
H	9.1238	−4.0448	−0.9204
H	7.6474	−3.4191	−0.1549
H	7.5387	−4.6751	−1.4007
C	8.6149	−3.1993	−3.4822
H	8.5738	−2.4339	−4.2643
H	9.6642	−3.4692	−3.3189
H	8.0911	−4.0881	−3.8478
C	5.5010	7.2114	2.2414
H	5.0388	6.9930	3.2102
H	6.1106	8.1136	2.3550
H	4.7070	7.4301	1.5201
C	7.0136	6.4391	0.4174
H	7.5884	7.3666	0.5181
H	7.6918	5.6603	0.0542
H	6.2360	6.5943	−0.3383
C	7.5022	5.8440	2.8057
H	8.0803	6.7666	2.9276
H	7.0800	5.5713	3.7787
H	8.1934	5.0524	2.5005
C	−7.0139	6.4387	−0.4171
H	−7.6920	5.6598	−0.0536
H	−6.2362	6.5940	0.3385
H	−7.5889	7.3661	−0.5177
C	−7.5030	5.8432	−2.8052
H	−8.1940	5.0516	−2.4998
H	−8.0813	6.7658	−2.9271
H	−7.0810	5.5705	−3.7783
C	−5.5019	7.2109	−2.2415
H	−6.1116	8.1131	−2.3550
H	−4.7077	7.4297	−1.5204
H	−5.0399	6.9925	−3.2104
O	4.9708	−3.7099	3.7591
C	6.0568	−2.8006	3.7786
H	6.0421	−2.1558	2.8921
H	6.0340	−2.1755	4.6793
H	6.9619	−3.4079	3.7846
O	3.4481	−6.5654	−0.1914
C	2.2809	−7.3046	−0.4876
H	1.5151	−7.1644	0.2854
H	1.8634	−7.0196	−1.4607
H	2.5802	−8.3520	−0.5159
O	−4.9708	−3.7099	−3.7593
C	−6.0568	−2.8006	−3.7789

H	−6.0421	−2.1558	−2.8924
H	−6.0338	−2.1755	−4.6796
H	−6.9619	−3.4079	−3.7850
O	−3.4473	−6.5655	0.1919
C	−2.2801	−7.3046	0.4885
H	−1.5143	−7.1645	−0.2845
H	−1.8628	−7.0193	1.4616
H	−2.5794	−8.3520	0.5169

R_2 Br^- double helix, E(RM062X) = −8089.89906987

H	−5.4007	4.2020	−0.1981
H	2.5945	6.0427	−0.0382
H	7.8317	−0.2133	−2.0390
H	−8.0744	1.1576	1.2148
H	2.5552	0.5311	−1.1102
C	8.5770	2.1733	−0.9294
C	−3.1946	3.0530	0.5149
C	−0.8732	4.3014	0.2642
C	0.4737	6.2893	0.2576
C	4.7334	1.7854	−0.9186
C	6.0223	2.3128	−0.7340
C	5.7137	−0.1925	−1.8966
H	−1.6630	6.3018	0.5346
C	−8.1300	3.6090	−0.0063
C	−6.0024	0.7680	1.4619
C	−7.0737	1.5526	1.0593
C	−6.9113	2.8018	0.4433
C	−5.6062	3.2417	0.2611
H	6.0925	3.2954	−0.2810
C	−4.4986	2.4820	0.6732
C	−2.1314	3.6279	0.3960
C	1.5260	4.1580	−0.0763
C	−0.7785	5.6968	0.3670
C	1.6313	5.5504	0.0382
C	0.2766	3.5464	0.0412
C	2.6685	3.3303	−0.3288
C	3.6030	2.5936	−0.5740
N	−3.5510	0.4873	1.6286
C	−4.6889	1.2175	1.2718
C	7.1638	1.6235	−1.1261
C	6.9701	0.3680	−1.7204
C	4.5689	0.5040	−1.4897
N	3.2708	−0.0052	−1.5949
H	−2.6673	0.8945	1.3323
H	−2.6944	−6.0774	0.0612
H	−8.0208	0.0429	−1.8901
H	5.2062	−4.3020	−0.1479
C	−4.8908	−1.8593	−0.7001

C	−6.1710	−2.3809	−0.4566
C	−7.3241	−1.7264	−0.8724
C	−7.1494	−0.5102	−1.5491
H	−6.2252	−3.3328	0.0594
C	−4.7432	−0.6197	−1.3592
C	5.9994	−0.8352	1.3464
C	6.7918	−2.9315	0.3629
C	0.7559	−4.3154	0.4159
C	0.6771	−5.7140	0.4526
C	−1.6444	−4.1812	0.1105
C	−2.7980	−3.3599	−0.1036
H	8.0430	−1.2996	1.0172
C	7.9623	−3.7881	−0.1209
C	−8.7286	−2.2790	−0.6243
H	1.5703	−6.3175	0.5701
C	4.4033	−2.5224	0.7011
C	7.0243	−1.6682	0.9246
C	5.4659	−3.3333	0.2644
C	4.6625	−1.2432	1.2376
C	3.0798	−3.0627	0.6142
C	2.0142	−3.6401	0.5325
C	−0.5703	−6.3142	0.3301
C	−1.7357	−5.5796	0.1567
C	−0.4042	−3.5593	0.2467
C	−3.7487	−2.6420	−0.3398
C	−5.8997	0.0435	−1.7884
Br	0.0229	0.0217	0.4524
H	5.6101	−1.1786	−2.3252
H	−6.1704	−0.2064	1.8963
H	−0.3403	−2.4798	0.2197
H	0.2025	2.4704	−0.0280
C	2.8659	−1.1223	−2.3100
O	3.6200	−1.8317	−2.9621
N	1.5101	−1.3099	−2.2340
H	1.0108	−0.7527	−1.5447
C	−3.5027	−0.6826	2.3713
O	−4.4929	−1.3126	2.7187
N	−2.2159	−1.0280	2.6997
H	−1.4785	−0.4422	2.3205
C	−1.7539	−2.3014	3.0911
C	−0.3821	−2.4242	3.3233
C	−2.5641	−3.4394	3.1971
C	0.1979	−3.6606	3.5902
C	−1.9840	−4.6727	3.4567
C	−0.6024	−4.8029	3.6235
H	0.2530	−1.5440	3.2548
H	−3.6295	−3.3599	3.0337
H	1.2713	−3.7171	3.7281

H	−2.5956	−5.5687	3.5012
C	0.7993	−2.4616	−2.6437
C	1.3737	−3.7335	−2.7481
C	−0.5724	−2.3154	−2.8467
C	0.5645	−4.8341	−2.9886
C	−1.3879	−3.4183	−3.0830
C	−0.8207	−4.6927	−3.1225
H	2.4387	−3.8566	−2.5981
H	−1.0140	−1.3223	−2.7995
H	0.9881	−5.8325	−3.0354
H	−2.4552	−3.2689	−3.1954
N	−3.4471	−0.1214	−1.5270
H	−2.7239	−0.6268	−1.0214
C	−3.0598	0.9732	−2.2826
N	−1.7059	1.1836	−2.2187
N	3.5748	−0.4396	1.5977
H	2.6626	−0.7604	1.2796
C	3.6331	0.7426	2.3200
N	2.4037	1.3245	2.4785
H	−5.8102	0.9969	−2.2882
O	−3.8257	1.6465	−2.9589
H	−1.1911	0.6709	−1.5063
C	−1.0148	2.3145	−2.7133
C	−1.6033	3.5754	−2.8656
C	0.3510	2.1685	−2.9518
C	−0.8133	4.6677	−3.1908
C	1.1486	3.2641	−3.2707
C	0.5685	4.5300	−3.3612
H	−2.6640	3.6977	−2.6883
H	0.8039	1.1830	−2.8683
H	−1.2484	5.6582	−3.2795
H	2.2128	3.1168	−3.4110
H	6.2245	0.1471	1.7335
O	4.6683	1.2011	2.7877
H	1.6165	0.8869	2.0062
C	2.1619	2.6547	2.8831
C	0.8369	3.0053	3.1383
C	3.1489	3.6487	2.9478
C	0.4731	4.3235	3.4070
C	2.7905	4.9584	3.2258
C	1.4536	5.3140	3.4342
H	0.0640	2.2418	3.0789
H	4.1819	3.3965	2.7538
H	−0.5732	4.5585	3.5640
H	3.5430	5.7402	3.2576
Cl	−0.6729	−8.0588	0.3935
Cl	0.5980	8.0267	0.4082
C	7.4977	−5.1453	−0.6582

H	8.3665	–5.7252	–0.9855
H	6.9769	–5.7248	0.1117
H	6.8283	–5.0311	–1.5174
C	8.6943	–3.0430	–1.2494
H	9.5350	–3.6420	–1.6171
H	8.0158	–2.8476	–2.0875
H	9.0917	–2.0839	–0.9006
C	8.9377	–4.0341	1.0420
H	8.4399	–4.5665	1.8593
H	9.7838	–4.6408	0.7010
H	9.3367	–3.0958	1.4399
C	9.3717	1.2146	–0.0272
H	8.8848	1.1080	0.9487
H	9.4574	0.2198	–0.4773
H	10.3854	1.5986	0.1332
C	9.2787	2.2868	–2.2928
H	10.2942	2.6768	–2.1621
H	9.3531	1.3145	–2.7899
H	8.7316	2.9666	–2.9543
C	8.5682	3.5568	–0.2717
H	8.0314	4.2897	–0.8835
H	8.1042	3.5278	0.7201
H	9.5970	3.9104	–0.1504
C	–9.4576	–2.4518	–1.9671
H	–8.9178	–3.1517	–2.6135
H	–10.4661	–2.8459	–1.7996
H	–9.5534	–1.5008	–2.5006
C	–9.5129	–1.2933	0.2568
H	–9.6020	–0.3128	–0.2227
H	–10.5250	–1.6711	0.4405
H	–9.0145	–1.1583	1.2232
C	–8.6950	–3.6361	0.0856
H	–8.2176	–3.5648	1.0689
H	–9.7181	–3.9948	0.2362
H	–8.1592	–4.3864	–0.5055
C	–8.8883	2.8172	–1.0843
H	–8.2420	2.6196	–1.9469
H	–9.2418	1.8566	–0.6956
H	–9.7611	3.3836	–1.4280
C	–9.0604	3.8526	1.1933
H	–9.9371	4.4290	0.8777
H	–9.4161	2.9128	1.6272
H	–8.5429	4.4155	1.9773
C	–7.7345	4.9677	–0.5934
H	–7.1931	5.5784	0.1371
H	–7.1068	4.8556	–1.4837
H	–8.6361	5.5147	–0.8868
O	–0.1327	–6.0692	3.8062

C	1.2675	–6.2185	3.9295
H	1.7866	–5.8118	3.0530
H	1.6430	–5.7204	4.8311
H	1.4563	–7.2895	4.0002
O	–1.5320	–5.8431	–3.2791
C	–2.9420	–5.7253	–3.2788
H	–3.2943	–5.2226	–2.3692
H	–3.2954	–5.1697	–4.1551
H	–3.3336	–6.7417	–3.3121
O	1.2626	5.6752	–3.6056
C	2.6736	5.5726	–3.6345
H	3.0542	5.1320	–2.7044
H	3.0122	4.9679	–4.4837
H	3.0517	6.5890	–3.7409
O	1.2106	6.6379	3.6416
C	–0.1353	7.0217	3.8416
H	–0.7542	6.7543	2.9770
H	–0.5516	6.5551	4.7422
H	–0.1294	8.1047	3.9615

REFERENCES

(1) M. P. Wintergerst, T. G. Levitskaia, B. A. Moyer, J. L. Sessler, and L. H. Delmau, *J. Am. Chem. Soc.* 2008, **130**, 4129–4139.

(2) B. W. Tresca, R. J. Hansen, C. V. Chau, B. P. Hay, L. N. Zakharov, M. M. Haley, and D. W. Johnson, *J. Am. Chem. Soc.* 2015, **137**, 14959–14967.

(3) P. Debye and E. Hückel, *Phys. Z.* 1923, **24**, 185–206.

(4) D. A. McQuarrie and J. D. Simon, in *Physical Chemistry: A Molecular Approach*, ed. A. McGuire, University Science Books, Sausalito, CA, 1997, Solutions II: Solid–Liquid Solutions, pp. 1011–1047.

(5) *Y.* Marcus, *Ion Properties*, Marcel Dekker, New York, NY, 1997.

(6) J. Rais, *Collect. Czech. Chem. Commun.* 1971, **36**, 3253–3262.

(7) Y. Marcus, *J. Soln. Chem.* 2008, **37**, 1071–1098.

(8) Y. Marcus, M. J. Kamlet and R. W. Taft, *J. Phys. Chem.*, 1988, **92**, 3613–3622.

(9) B. Hundhammer, and S. Wilke, *J. Electroanal. Chem.* 1989, **266**, 133–144.

(10) C. F. Baes Jr., *SXLSQI, A Program for Modeling Solvent Extraction Systems*, ORNL/TM–13604, Oak Ridge National Laboratory, Oak Ridge, TN, 1998.

(11) Y. Deng, R. A. Sachleben, and B. A. Moyer, *J. Chem. Soci., Faraday Trans.* 1995, **91**, 4215–4222.

(12) C. F. Baes Jr., *Solvent Extr. Ion Exch.* 2001, **19**, 193–213.

(13) F. J. Millero, in *Water and Aqueous Solutions: Structure, Thermodynamics, and Transport Processes*, ed. R. A. Horne, Wiley–Interscience, NY, 1972, The Partial Molal Volumes of Electrolytes in Aqueous Solutions, p. 572.

(14) A. F. M. Barton, *Handbook of Solubility Parameters and Other Cohesion Parameters*, CRC Press, Boca Raton, FL, 1991.

(15) C. F. Baes Jr. and B. A. Moyer, *J. Phys. Chem. B* 1997, **101**, 6566–6574.

(16) K. Pitzer, in *Activity coefficients in electrolyte solutions,* CRC Press, Boca Raton, FL, 2nd edn., 1991, Ion Interaction Approach: Theory and Data Correlation, p.100.
(17) J. A. Riddick, W. B. Bunger, and T. K. Sakano, *Organic Solvents: Physical Properties and Methods of Purification*, Wiley–Interscience, NY, 1986.
(18) Gaussian 16, Revision C.02, M. J. Frisch, G. W. Trucks, H. B. Schlegel, G. E. Scuseria, M. A. Robb, J. R. Cheeseman, G. Scalmani, V. Barone, G. A. Petersson, H. Nakatsuji, X. Li, M. Caricato, A. V. Marenich, J. Bloino, B. G. Janesko, R. Gomperts, B. Mennucci, H. P. Hratchian, J. V. Ortiz, A. F. Izmaylov, J. L. Sonnenberg, D. Williams–Young, F. Ding, F. Lipparini, F. Egidi, J. Goings, B. Peng, A. Petrone, T. Henderson, D. Ranasinghe, V. G. Zakrzewski, J. Gao, N. Rega, G. Zheng, W. Liang, M. Hada, M. Ehara, K. Toyota, R. Fukuda, J. Hasegawa, M. Ishida, T. Nakajima, Y. Honda, O. Kitao, H. Nakai, T. Vreven, K. Throssell, J. A. Montgomery, Jr., J. E. Peralta, F. Ogliaro, M. J. Bearpark, J. J. Heyd, E. N. Brothers, K. N. Kudin, V. N. Staroverov, T. A. Keith, R. Kobayashi, J. Normand, K. Raghavachari, A. P. Rendell, J. C. Burant, S. S. Iyengar, J. Tomasi, M. Cossi, J. M. Millam, M. Klene, C. Adamo, R. Cammi, J. W. Ochterski, R. L. Martin, K. Morokuma, O. Farkas, J. B. Foresman, and D. J. Fox, Gaussian, Inc., Wallingford CT, 2016.
(19) Y. Zhao and D. G. Truhlar, *Theor. Chem Account* 2008, **120**, 215–241.
(20) R. F. Ribeiro, A. V. Marenich, C. J. Cramer and D. G. Truhlar, *J. Phys. Chem. B* 2011**, 115**, 14556–14562.
(21) M. Cossi, V. Barone, R. Cammi, and J. Tomasi, *Chem. Phys. Lett.* 1996, **255**, 327–335.
(22) B. Mennucci and J. Tomasi, *J. Chem. Phys.* 1997, **106,** 5151–5158.
(23) C. A. Johnson, O. B. Berryman, A. C. Sather, L. N. Zakharov, M. M. Haley and D. W. Johnson, *Cryst. Growth Des.* 2009, **9**, 4247–4249.
(24) B. W. Tresca, R. J. Hansen, C. V. Chau, B. P. Hay, L. N. Zakharov, M. M. Haley and D. W. Johnson. *J. Am. Chem. Soc.* 2015, **137**, 14959–14967.
